# The CDK12–BRCA1 signaling axis mediates dinaciclib‐associated radiosensitivity through p53‐mediated cellular senescence

**DOI:** 10.1002/1878-0261.13773

**Published:** 2024-12-03

**Authors:** Natalia García Flores, Diego M. Fernández‐Aroca, Cristina Garnés‐García, Andrés Domínguez‐Calvo, Jaime Jiménez‐Suárez, Sebastià Sabater, Pablo Fernández‐Aroca, Ignacio Andrés, Francisco J. Cimas, Guillermo de Cárcer, Borja Belandia, Ignacio Palmero, Pablo Huertas, María José Ruiz‐Hidalgo, Ricardo Sánchez‐Prieto

**Affiliations:** ^1^ Laboratorio de Oncología Molecular, Unidad de Medicina Molecular, Instituto de Biomedicina Universidad de Castilla‐La Mancha Albacete Spain; ^2^ Unidad de Biomedicina de la UCLM, Unidad asociada al CSIC Albacete Spain; ^3^ Centre for Genomics and Child Health, Blizard Institute, Faculty of Medicine and Dentistry Queen Mary University of London UK; ^4^ Translational Cancer Research Group, Chronic Diseases and Cancer, Area 3, Instituto Ramón y Cajal de Investigación Sanitaria (IRYCIS) Madrid Spain; ^5^ Facultad de Biología Universidad de Sevilla Spain; ^6^ Centro Andaluz de Biología Molecular y Medicina Regenerativa‐CABIMER Universidad de Sevilla‐CSIC‐Universidad Pablo de Olavide Spain; ^7^ Servicio de Oncología Radioterápica Complejo Hospitalario Universitario de Albacete Spain; ^8^ Área de Bioquímica y Biología Molecular, Facultad de Medicina Universidad de Castilla‐La Mancha Albacete Spain; ^9^ Departamento de Biología del Cáncer Instituto de Investigaciones Biomédicas Sols‐Morreale (CSIC‐UAM) Madrid España; ^10^ CSIC Conexión‐Cáncer Hub Madrid Spain; ^11^ Laboratorio de Senescencia Celular y Supresión Tumoral, Departamento de Biología del Cáncer Instituto de Investigaciones Biomédicas Sols‐Morreale (CSIC‐UAM) Madrid Spain

**Keywords:** BRCA1, CDK12, dinaciclib, radiosensitivity, senescence

## Abstract

Pan‐cyclin‐dependent‐kinase (CDK) inhibitors are a new class of targeted therapies that can act on multiple CDKs, with dinaciclib being one of the most promising compounds. Although used as a monotherapy, an interesting approach could be to combine it with radiotherapy. Here, we show that dinaciclib increases radiosensitivity in some experimental models of lung and colon cancer (A549 or HCT 116) but not in others (H1299 or HT‐29). Dinaciclib did not alter serine‐protein kinase ATM signalling or cell cycle profiling after ionising‐radiation exposure, which have been described for other CDK inhibitors. Interestingly, in terms of apoptosis, although the combination renders a clear increase, no potentiation of the ionising‐radiation‐induced apoptosis was observed. Mechanistically, inhibition of CDK12 by dinaciclib diminishes *BRCA1* expression, which decreases homologous recombination (HR) and probably promotes the nonhomologous end joining repair process (NHEJ), which ultimately promotes the induction of ionising‐radiation‐associated cellular senescence in a *TP53*‐dependent manner, explaining the lack of effect observed in some experimental models. In conclusion, our report proposes a molecular mechanism, based on the signalling axis CDK12–BRCA1, involved in this newly identified therapeutic effect of dinaciclib, although other players implicated in HR should not be discarded. In addition, our data provide a rationale for more selective and personalised chemo/radiotherapy treatment according to the genetic background of the tumour.

## Graphical abstract

Dinaciclib blocks CDK12 activity, leading to downregulation of BRCA1, and probably other proteins involved in homologous recombination, promoting nonhomologous end joining. In this scenario, the presence of ionising radiation triggers a p53‐dependent senescent response that yields increased radiosensitivity.

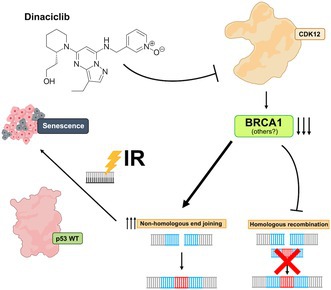

**Abbreviations**
CDKs: cyclin‐dependent kinases
HR: homologous recombination
IR: ionising radiation
NHEJ: nonhomologous end joining
SASP: senescence‐associated secretory phenotype
SA‐β‐Gal: senescence‐associated β‐galactosidase
SD: standard deviation
SF: surviving fraction
shRNA: short hairpin RNA
TGCA: The Cancer Genome Atlas
WT: wild type

## 1 Introduction

The cell cycle has become one of the major targets in cancer therapy. Indeed, in recent years, there have been significant advances in targeting the cellular machinery of the cell cycle, with a particular focus on cyclin‐dependent kinases (CDKs) (for a review, see Ref. [1]). In fact, CDK4/6 inhibitors are currently used in clinical practice to treat metastatic ER+ breast cancer [1] and are also being considered for use in other cancers [2]. In addition to CDK4/6 inhibitors, a new class of CDK inhibitors has been proposed with a broader spectrum of activity, acting on multiple CDKs simultaneously, of which dinaciclib is a paradigmatic example. This novel pan‐CDK inhibitor has a preferential effect on CDK1, 2, 5, and 9 with a lower affinity for CDK4 and CDK6 [3]. In addition, recent evidence has shown that CDK12 is a novel target for dinaciclib [4], increasing its potential in cancer therapy [5]. In fact, clinical trials are underway to evaluate the use of dinaciclib in various tumour types [6, 7]. In addition, to a clear antitumour effect in various experimental models, from osteosarcoma [8] to cholangiocarcinoma [9], dinaciclib has shown an exceptional ability to enhance the efficacy of conventional chemotherapy [10, 11, 12] or novel therapeutic approaches [13]. Interestingly, there is increasing evidence linking dinaciclib to the DNA repair machinery through effects on molecules such as BRCA1 [4, 10, 14]. In fact, recent evidenced proposes the use of dinaciclib as a radiosensitizer in cervical cancer cell lines [15], but the molecular basis for this effect remains unexplored.

With this in mind, we decided to evaluate the potential of dinaciclib as a radiosensitizing agent. Our data show that dinaciclib exerts a potent radiosensitizing effect in various experimental models of colon and lung cancer by promoting ionising radiation (IR)‐dependent senescence. This effect is the consequence of CDK12 inhibition, which among other effects, lead to *BRCA1* downregulation, affecting HR, a perfect context for triggering a *TP53*‐dependent IR‐associated senescence.

In conclusion, our data demonstrate the potential of dinaciclib in combination with radiotherapy and describe one of the signalling axis that mediates this effect, allowing the correct selection of patients who could benefit from this therapeutic combination.

## 2 Materials and methods

### 2.1 Plasmids used for short hairpin RNA (shRNA) interference were as cell lines, plasmids, chemicals, and antibodies

A549 (RRID: CVCL_0023), H1299 (RRID: CVCL_0060), HCT 116 (RRID: CVCL_UR40), HT‐29 (RRID: CVCL_0320), HEK293T (RRID: CVCL_0063), and U2OS (RRID: CVCL_0042) cells have been obtained from ATCC (LGC Standards S.L.U., Barcelona, Spain) and cultured as previously described [16]. HCT 116 +/+ and −/− were provided by B. Vogelstein (Johns Hopkins University School of Medicine, Baltimore, Maryland, USA). Cells were maintained in 5% CO_2_ and 37 °C; and grown in Dulbecco's modified Eagle's medium supplemented with 10% fetal bovine serum, 1% glutamine, and 1% penicillin/streptomycin. All cell culture reagents were provided by Lonza (Culteck S.L., Madrid, Spain). Cultures were periodically tested for mycoplasma contamination and authenticated by the GenePrint® 10 System (Promega, Madison, WI, USA), and data were analysed using the genemapper® id‐x v1.2 software (Applied Biosystems, Waltham, MA, USA) at the genomic core facility of the Instituto de Investigaciones Biomedicas Sols‐Morreale.

Plasmids used for short hairpin RNA (shRNA) interference were as follows: Human PLKO.1‐puro‐shRNAp53 and Empty vector has been previously described [16]. pLKO.1‐puro‐shRNACDK12 (TRCN0000196423 and TRCN0000368335), and pLKO.1‐puro‐shRNABRCA1 (TRCN0000244984 and TRCN0000244987) were purchased from Merck (Madrid, Spain).

Dinaciclib (MedChemExpress, EURODIAGNOSTICO S.L., Madrid, Spain), navitoclax (MedChemExpress), and ATM inhibitor Ku‐55 933 (Calbiochem, Merck, Madrid, Spain) were dissolved in DMSO, aliquoted, and stored at −80 °C. Puromycin (Merck) were dissolved in double‐distilled water, aliquoted, and stored at −20 °C.

Antibodies used for western blot or immunofluorescence are summarised in Table S1.

### 2.2 Transfections and infections

Lentiviral production and cell infection were performed as previously described [16]. In all knockdown experiments, infected cells were selected with 1 μg·mL^−1^ puromycin for 72 h. Each experiment was performed with three different pools of infection. Infected cells were maintained for a maximum of 2 weeks after selection and then discarded and replaced with a new fresh pool of infected cells.

### 2.3 Western blotting

Cells were collected in RIPA lysis buffer (50 mm Tris, pH 8; 1.5 mm MgCl_2_; 1 mm EDTA; 1 mm EGTA; 1% Triton X‐100; 150 mm NaCl; 20 mm β‐glycerophosphate; 0.1% SDS; 0.5% desoxycholic acid). Protease and phosphatase inhibitors (Merck) were added prior to lysis. Protein quantification and western blotting was performed as previously described [17]. One hundred microgram, otherwise is indicated, of protein was loaded onto appropriate percentage SDS/PAGE, transferred to PVDF membranes using semidry Pierce Power Blot (Thermo Fisher Scientific, Alcobendas, Madrid, Spain) and blotted against different proteins via specific antibodies. Antibodies were detected by enhanced chemiluminescence (Amersham, Thermo Fisher Scientific) in a LAS‐3000 system (FujiFilm, Alcobendas, Madrid, Spain).

### 2.4 Immunocytochemistry

Cells were grown onto SPL cell culture slides (Labclinic, Barcelona, Spain) prior to IR. After treatment cells were fixed, permeabilized, and incubated with the indicated antibodies as previously described [18]. Positive immunofluorescence was detected using a Zeiss (Tres Cantos, Madrid, Spain) Apotome fluorescence microscope and processed using Zen 2009 Light Edition program (Zeiss). Foci quantification was performed with CellProfiler (Broad Institute Cambridge, MA, USA) [19]. Images show a representative cell from a minimum of 100 quantified (5 fields per sample captured). Data shown are the average of, at least, three independent experiments.

### 2.5 RNA isolation, reverse transcription, and real‐time quantitative PCR

Total RNA obtention, cDNA synthesis, and real‐time PCR were performed as previously described [20]. Primers for all target sequences were designed by using the NCBI blast software and purchased from Merck as DNA oligos. Primer sequences can be found in Table S2. Data shown are the average of, at least, three independent experiments performed in triplicate.

### 2.6 Irradiation and clonogenic assays

Cells were irradiated by the technical staff of Radiotherapy Unit at University General Hospital of Albacete, in a Clinac Low Energy 600C linear electron accelerator from Varian (Palo Alto, CA, USA) at a dose rate of 600 cGy·min^−1^ in a radiation field of 40 × 40 cm. Cells were plated and the following day, cells were treated with 10 nm dinaciclib for 24 h prior to IR. Then, fresh medium was added, and cells were incubated at 37 °C for 10–14 days to allow colony formation. Clonogenic assays were performed and valuated as previously described [20, 21]. Values were referred to unirradiated controls, set at 1. SF2Gy was calculated by applying a linear‐quadratic model [22]. Data shown are the average of, at least, three independent experiments performed in triplicated cultures.

### 2.7 Senescence‐associated β‐galactosidase activity (SA‐β‐Gal)

Six days after IR, cells were washed in PBS, fixed for 5 min (room temperature) in 2% formaldehyde, 0.2% glutaraldehyde, washed twice with PBS for 5 min, and incubated for 16 h at 37 °C with freshly prepared SA‐β‐Gal staining solution as previously described [23]. After this, cells were washed twice with PBS for 5 min. Images were acquired at 20× and show a representative field out of five acquired per sample (minimum of 100 cells quantified per condition). Data shown are the average of three independent experiments.

### 2.8 Flow cytometry

For cell cycle analysis, 10^5^ cells were seeded in 6‐cm plates. Twenty‐four h later, cells were exposed to different treatments. Cell cycle was analysed as previously described at indicated times after IR treatment [16]. Apoptosis, usually 48 h after IR exposure, was detected with Annexin V‐FITC (Immunostep) following the manufacturer's instructions. Samples and data were processed in a MACSQuant Analyzer 10 (Miltenyi Biotec S.L., Pozuleo de Alarcaon, Madrid, Spian). Data shown are the average of, at least, three independent experiments performed.

### 2.9 Dose–response measurements

For cell proliferation measurements, 10^4^ cells/well were seeded in 24‐well plates and proliferation was analysed 16 h and 3 days after treatment by an MTT‐based assay as previously described [17]. Data shown are the average of three independent experiments performed in triplicated cultures.

### 2.10 GFP reporter assay

U2OS cells harbouring a single copy of the reporter constructs DR‐GFP (HR [24]) or EJ5‐GFP (NHEJ [25]) were used as previously described [26]. They were grown in a standard medium supplemented with 1 μg·mL^−1^ puromycin. Briefly, 60 000 cells were plated in six‐well plates in duplicate. One day after seeding, cells were treated with dinaciclib (10 nm) or mock treated. The next day, each duplicate culture was infected with lentiviral particles containing I‐SceI–BFP expression construct at MOI 10 using 8 μg·mL^−1^ polybrene in 2 mL of DMEM. Then, cells were left to grow for an additional 24 h before changing the medium for fresh DMEM. Forty‐eight h after viral transduction, cells were trypsinized after a PBS wash and resuspended in 1 mL of PBS. Samples were analysed with a LSRFortessa™ Cell Analyzer (BD, San Agustin de Guadalix, Madrid, España) Flow Cytometer.

### 2.11 Statistical analysis

Data are presented as mean ± standard deviation (SD). Statistical significance was evaluated by Student's *t*‐test or ANOVA using the graphpad prism v9.0 software (Dotmatic, Boston, MA, USA). The statistical significance of differences is indicated in figures by asterisks as follows: *P < 0.05, **P < 0.01 and ***P < 0.001.

## 3 Results

### 3.1 Dinaciclib is a radiosensitizing agent that does not affect ATM signalling, apoptosis or cell cycle in response to IR

**Fig. 1:**
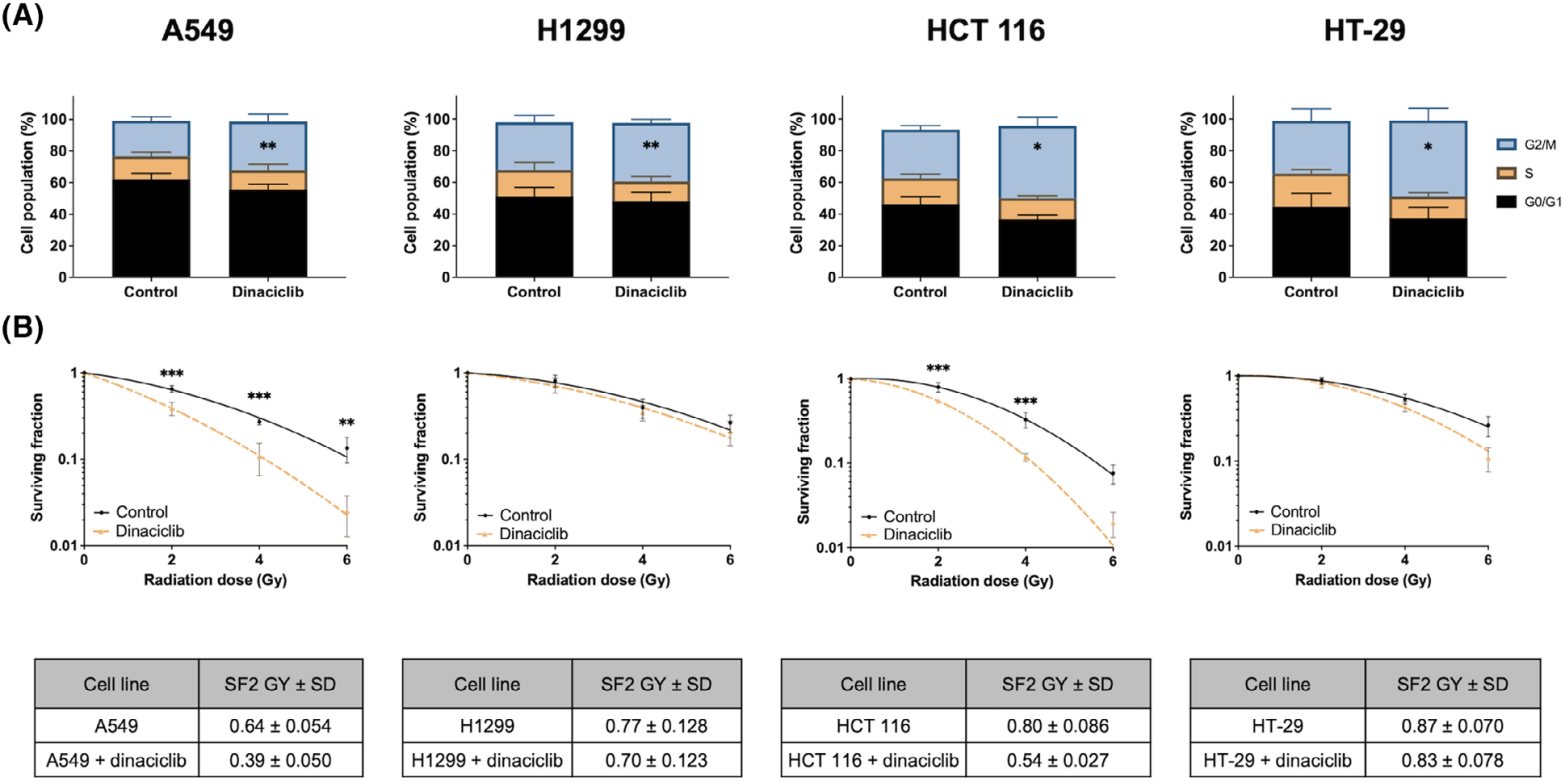
Dinaciclib promotes radiosensitivity in A549 and HCT 116 but not in H1299 or HT‐29 cell lines. (A) A549, H1299, HCT 116 and HT‐29 cell lines were incubated for 24 h in the presence of vehicle (DMSO) or dinaciclib (10 nm) and then, cell cycle was analysed. Histograms represent the average of three independent experiment. Bars denote standard deviation (SD). The unpaired Student's *t*‐test was used to assess statistical significance. (B) Upper panels: Clonogenic assays for A549, H1299, HCT 116 and HT‐29. Cells were plated and 24 h later exposed to vehicle or dinaciclib (10 nm) for additional 24 h. Then, cells were exposed to the indicated doses of X rays and immediately after media was replaced. Graphics shows represent the average of four independent experiments performed in triplicated cultures. Bars mean SD. Curves were fitted using lineal‐quadratic model. The two‐way ANOVA test was used to assess statistical significance. Lower panels: Surviving fraction was normalised to respective unirradiated controls. The statistical significance of differences is as follows: *P < 0.05, **P < 0.01 and ***P < 0.001.

First, we analysed the toxicity associated with dinaciclib in different experimental model at different doses. As it showed (Fig. S1A), all cell lines showed a similar toxicity being slightly lower in HT‐29, but in all cases with an IC50 between 5 and 10 nm. In addition, the distribution of the cell cycle was analysed after 24 h of incubation (10 nm), showing alterations of the cell cycle in all cell lines (Fig. 1A, Fig. S2A). Next, we analysed the combination of a 24‐h preincubation with 10 nm dinaciclib, with a toxicity lower than 20% in any of the experimental models (Fig. S1B), and IR by clonogenic assay. As shown in Fig. 1B, 24‐h pretreatment with dinaciclib 10 nm enhanced radiosensitivity in A549 and HCT 116 but failed in H1299 and HT‐29. Of note, A549 and HCT 116 cells have a WT *p53* that is functional in response to IR (Fig. S3), while H1299 and HT‐29 cells have no functional p53 due to homozygotic deletion or mutation, respectively [27]. Therefore, the radiosensitizing potential of dinaciclib shows an apparent correlation with the presence of a wild‐type (WT) *TP53*.

**Fig. 2:**
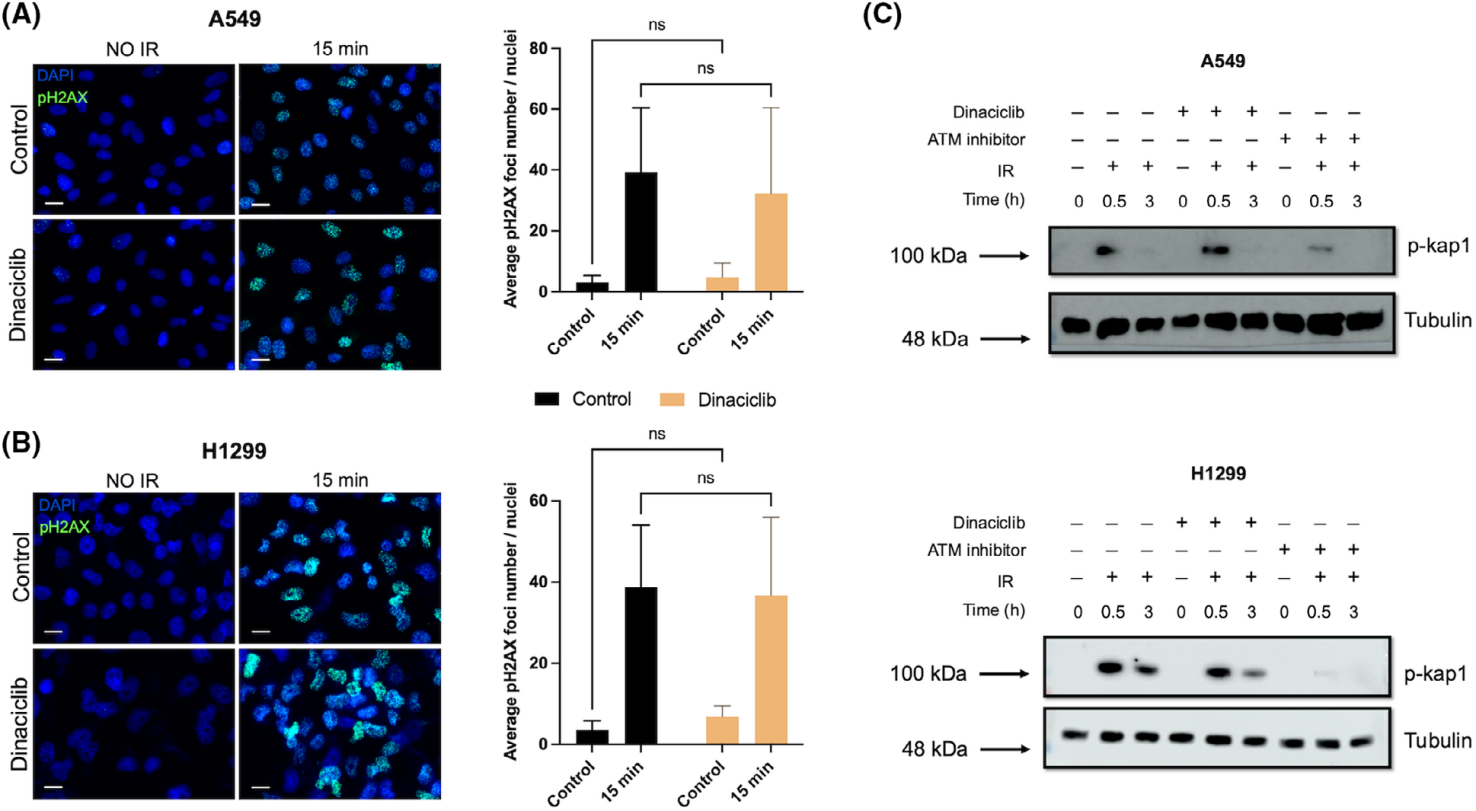
ATM signalling is not targeted by dinaciclib. (A) A549 (left panel) cells were plated onto cell culture slides and 24 h later exposed to vehicle or dinaciclib (10 nm) for additional 24 h. After irradiation (10 Gy) cells were fixed and processed for immunocytochemistry against phospho‐Histone H2AX (Ser139). Images show a representative field out of five analysed per condition. Scale bars represent 20 μm. Quantification of phospho‐Histone H2AX (right panel) average foci number per nuclei in three independent experiments. Bars mean SD. The two‐way ANOVA test was used to assess statistical significance. ns, not significative. (B) Same as in A for H1299 cells. (C) KAP1 phosphorylation was evaluated in A549 (upper panel) and H1299 (lower panel) cell lines in presence/absence of dinaciclib by western blot at indicated times after exposure to 10 Gy. Cells were treated with vehicle or dinaciclib (10 nm) or ATM inhibitor (10 μm) for 24 h before ionising radiation exposure. Protein extracts were blotted against the indicated antibodies. Image shows a representative experiment out of 3, with nearly identical result.

**Fig. 3:**
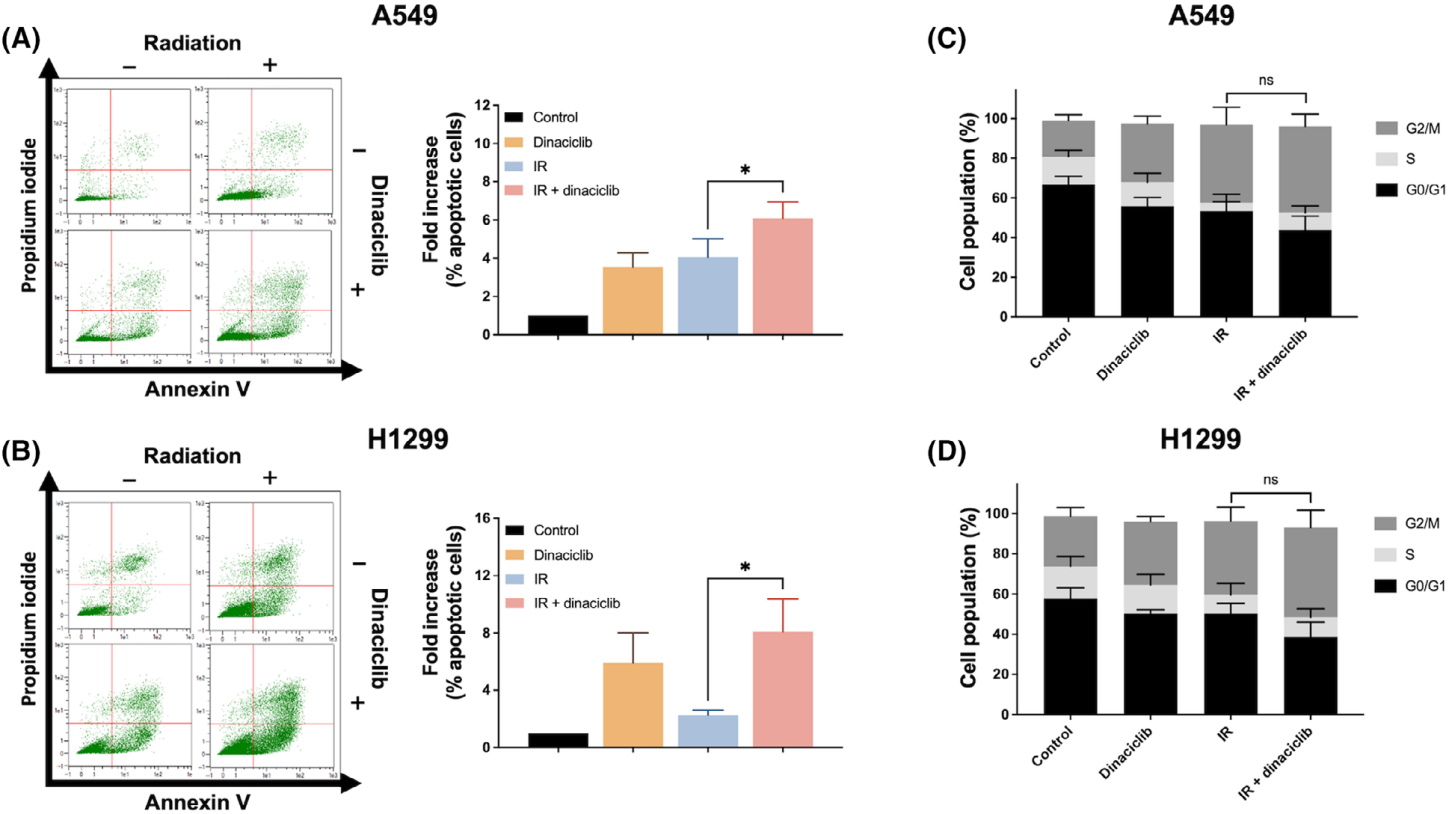
Dinaciclib does not modulates apoptosis or cell cycle in response to ionising radiation. (A) Left panel: Representative images of apoptosis induction in one out three independent experiments 48 h after irradiation (10 Gy) in A549 cells pretreated with dinaciclib (10 nm) or vehicle for 24 h prior to irradiation. Right panel: Histogram showing the average of three independent experiments. Bars mean SD. The unpaired Student's *t*‐test was used to assess statistical significance. (B) Same as in A for H1299 cells. (C) Cell cycle was evaluated by flow cytometry 48 h after ionising radiaton exposure (10 Gy) in A549 cells treated as in A. Histogram showing the average of three independent experiments representing the percentage of population in the different phases of the cell cycle. Bars mean SD. The unpaired Student's *t*‐test was used to assess statistical significance. (D) Same as in C for H1299 cells. The statistical significance of differences is indicated in figures by asterisks as follows: *P < 0.05. ns, not significative.

In view of our previous findings, we carried out an in‐depth analysis of the putative mechanism by which dinaciclib promotes radiosensitivity focussing on two cell lines, A549 and H1299. First, we analysed whether dinaciclib affects ATM activity. Therefore, we irradiated cells in the presence/absence of dinaciclib pretreatment and evaluated ATM signalling. As it shown in Fig. 2A–C, neither H2AX nor KAP1 phosphorylation after IR exposure were affected by the presence of dinaciclib. Next, we evaluated apoptosis after IR exposure. As shown in Fig. 3A,B, 48 h after IR exposure, dinaciclib did not show a synergistic effect in terms of IR‐induced apoptosis, being at best additive, and with identical behaviour regardless of the radiosensitizing effect. In fact, similar result was obtained in HCT 116 and HT‐29 experimental model (Fig. S4). In addition, cell cycle profiles were also evaluated 48 h after IR. Interestingly, irradiated cells pretreated with dinaciclib, showed a similar pattern to untreated cells (Fig. 3C,D, Fig. S2B), and again this observation was almost identical regardless of the radiosensitizing effect observed.

In conclusion, this series of experiments discards ATM signalling, apoptosis, or gross cell cycle alterations as the putative mechanisms of dinaciclib‐associated radiosensitivity in our experimental model.

### 3.2 Dinaciclib promotes senescence associated with IR in a p53‐dependent fashion

**Fig. 4:**
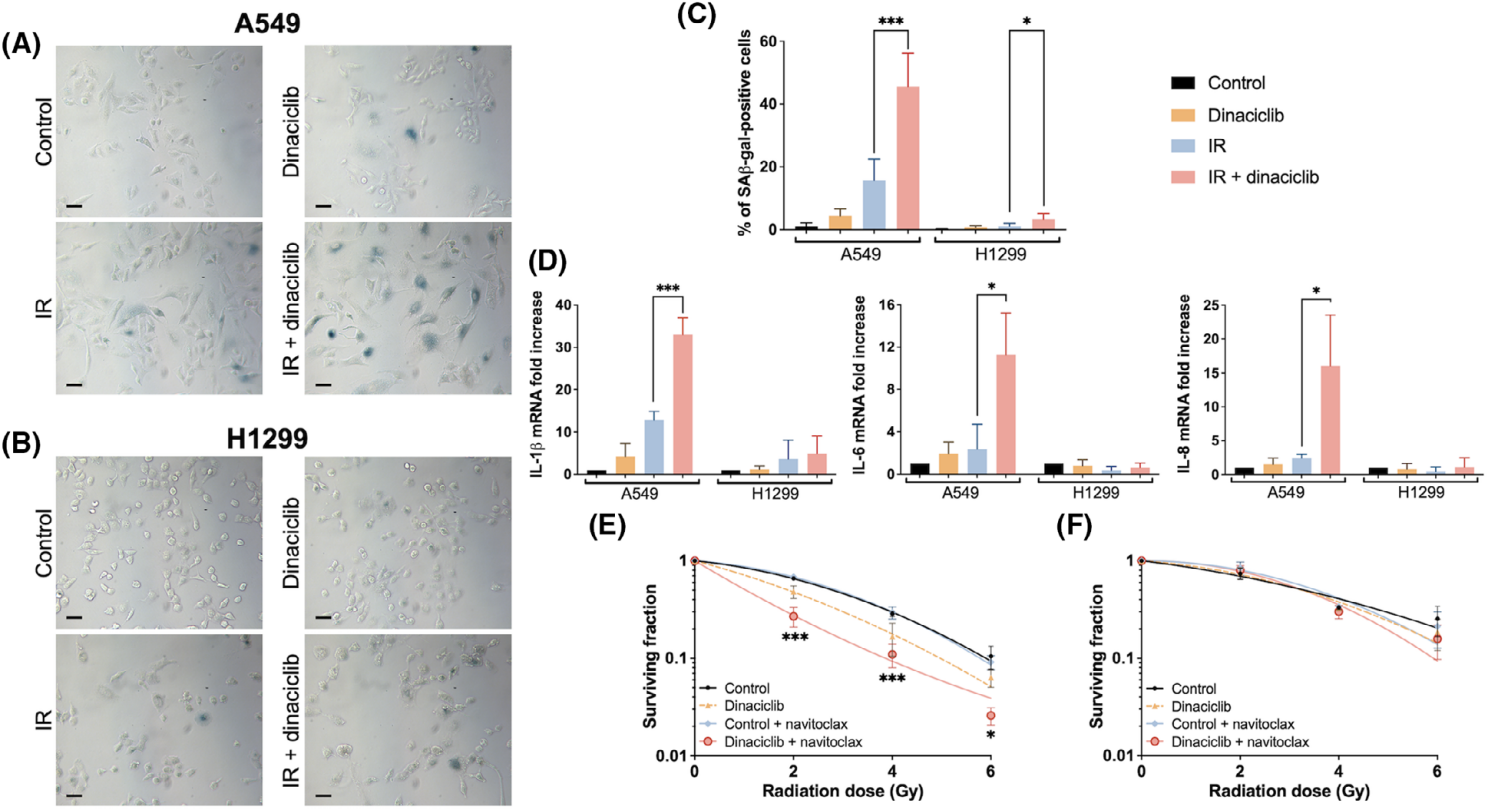
Dinaciclib enhances cell senescence in response to ionising radiation. (A) A549 cells pretreated with dinaciclib (10 nm) or vehicle for 24 h were irradiated (6 Gy) and 6 days later SA‐β‐Gal activity was detected by X‐Gal staining. Images show a representative field out of a minimum of five analysed per condition in four independent experiments. Scale bars represent 100 μm. (B) Same as in (A) for H1299 cells. (C) Histogram showing the average of four independent experiments representing the percentage of positive senescent cells in A549 and H1299 cell lines. The unpaired Student's *t*‐test was used to assess statistical significance. Bars mean SD. (D) Gene expression of indicated SASP genes in different conditions was evaluated 6 days after ionising radiation exposure (6 Gy) in A549 and H1299 cells by RT‐qPCR using GAPDH as an endogenous control. Data were referred to unirradiated and untreated cells (control). Histogram shows the average of three independent experiments. The unpaired Student's *t*‐test was used to assess statistical significance. Bars mean SD. A549 (E) and H1299 (F) cells pretreated as in A and then exposed or not to navitoclax (1 μm) for 72 h starting at day 5 after ionising radiation exposure. Then, media were replaced with navitoclax‐free medium until the end of the experiment. Surviving fraction was normalised to respective unirradiated controls or unirradiated pretreated cells with navitoclax. Curves were fitted using lineal‐quadratic model. Graphics represent the average of three independent experiment performed in triplicated cultures. The two‐way ANOVA test was used to assess statistical significance. Bars mean SD. The statistical significance of differences is indicated in figures by asterisks as follows: *P < 0.05 and ***P < 0.001.

All of the above suggests that other events may be involved in our initial observation. Therefore, we decided to investigate IR‐associated senescence. Interestingly, SA‐β‐Gal staining in the A549 cell line 6 days after IR exposure was significantly increased in the presence of dinaciclib pretreatment compared with separate treatments (Fig. 4A,C), showing a synergistic effect (> 2.5‐fold increase). However, SA‐β‐Gal staining was barely detected in H1299 cell line (Fig. 4B,C), suggesting a possible mechanism of radiosensitivity. Indeed, similar results were obtained in the HCT 116 and HT‐29 cell lines (Fig. S5). To support this observation, we analysed several genes associated with the Senescence‐Associated Secretory Phenotype (SASP) by RT‐qPCR, showing an enhanced expression in the A549 cells but not in H1299 cells, a result fully consistent with SA‐β‐Gal staining (Fig. 4D). Furthermore, p21/WAF expression was analysed in the same conditions as SA‐β‐gal staining showing a result fully consistent with our observations in terms of cellular senescence (Fig. S6). Next, we decided to use navitoclax, a known senolytic compound [28]. Based on our previous experiments, we incubated cells with navitoclax (1 μm) for 48 h, 5 days after IR exposure. Interestingly, navitoclax was able to promote a marked increase in radiosensitivity associated with dinaciclib pretreatment in our experimental model of A549 but not in H1299 cells (Fig. 4E,F). Indeed, in this experimental setting, SA‐β‐Gal staining was performed showing a fully concordant result with the senolytic activity of navitoclax (Fig. S7).

**Fig. 5:**
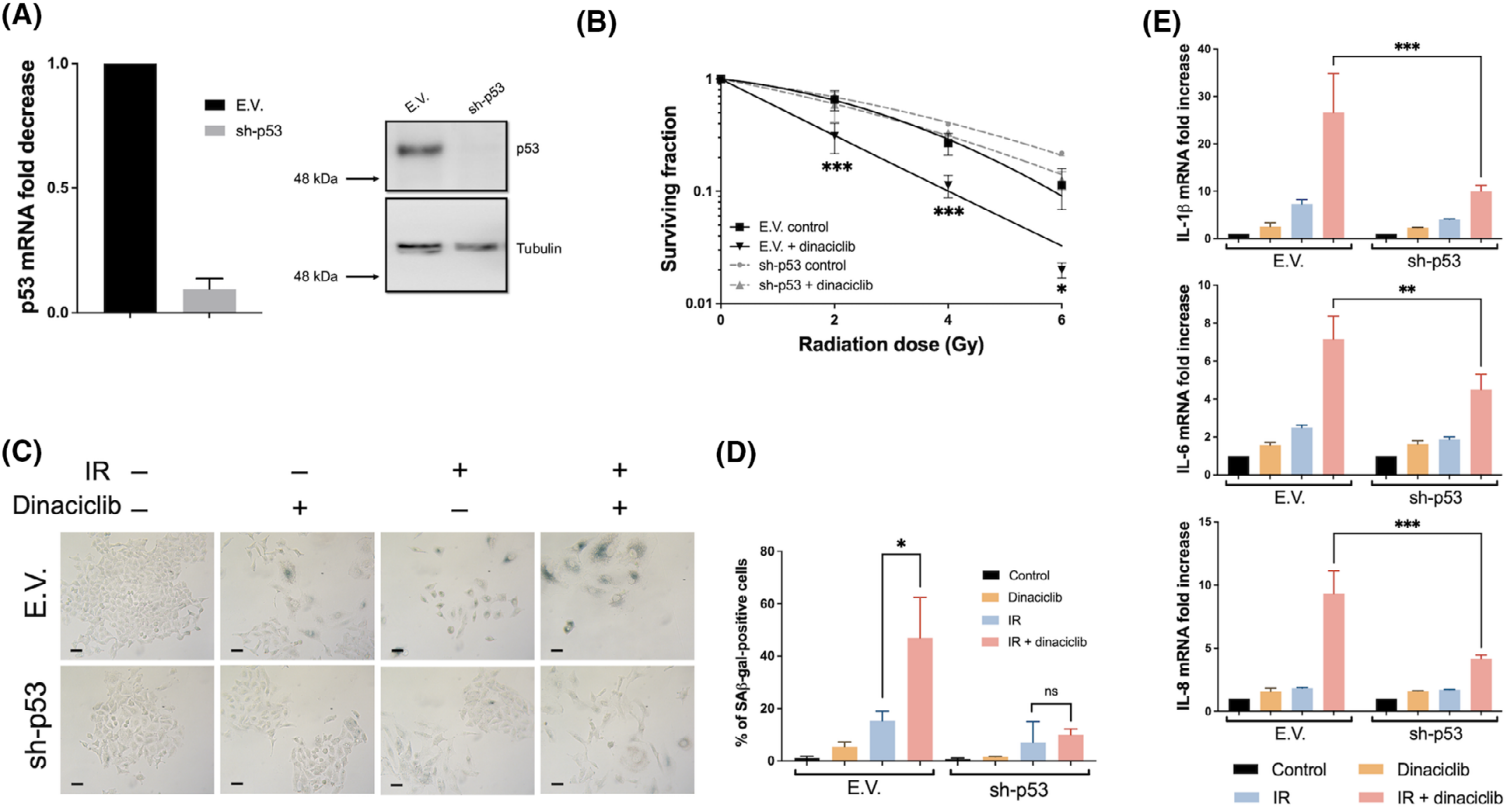
p53 is required for dinaciclib associated radiosensitivity. (A) A549 cells were infected with lentiviruses carrying empty vector (E.V.) or shRNA for p53 (sh‐p53). Selected pools were evaluated by RT‐qPCR (left panel average of three independent infections performed in triplicate) and by western blot using tubulin as a loading control (right panel representative experiment out of 3). Bars mean SD. (B) A549 cells infected with lentiviruses carrying E.V. or sh‐p53 were plated and 24 h later exposed to vehicle or dinaciclib (10 nm) for additional 24 h. Then, cells were exposed to the indicated doses of X rays and immediately after media was replaced. Cellular radiosensitivity was plotted using respective control cells. Curves were fitted using lineal‐quadratic model. Graphics represent the average of three independent experiment performed (one per infection) in triplicated cultures. The two‐way ANOVA test was used to assess statistical significance. Bars mean SD. (C): E.V. (upper panels) or sh‐p53 (lower panels) cells were irradiated (6 Gy) in presence/absence of dinaciclib 24 h pretreatment (10 nm) and 6 days later β‐Gal activity was detected by X‐gal staining. Images show a representative field out of five analysed per condition in three independent experiments. Scale bars represent 100 μm. (D) Histogram showing the average of three independent experiments representing the percentage of positive cells. The unpaired Student's *t*‐test was used to assess statistical significance. Bars mean SD. (E) Gene expression of indicated SASP genes in different conditions was evaluated 6 days after ionising radiation exposure (6 Gy) in E.V. or sh‐p53 cells by RT‐qPCR using GAPDH as an endogenous control. Data were referred to unirradiated and untreated cells (control). Histogram shows the average of three independent experiments. The two‐way ANOVA test was used to assess statistical significance. Bars mean SD. The statistical significance of differences is indicated in figures by asterisks as follows: *P < 0.05, **P < 0.01 and ***P < 0.001.

Our initial observation showed that in cells with null or dysfunctional *TP53* gene, dinaciclib is unable to promote radiosensitivity, so we decided to test the role of *TP53* in the radiosensitizing effect of dinaciclib. To this end, we knocked down p53 expression in A549 cells using a specific shRNA. As shown, after effective knockdown of *TP53* expression (Fig. 5A), the radiosensitizing effect of dinaciclib was drastically reduced, correlating with a lack of senescence (Fig. 5B–E). Furthermore, in the isogenic cell line model HCT 116 p53 null (HCT 116 p53 +/+ and −/−), we also observed a clear decrease in the radiosensitizing effect of dinaciclib as well as SA‐β‐gal staining in IR‐exposed cells after pretreatment with dinaciclib (Fig. S8). Collectively, these data support that p53‐mediated radiation‐associated senescence is an important determinant of the radiosensitizing effect of dinaciclib.

### 3.3 Dinaciclib blocks HR

**Fig. 6:**
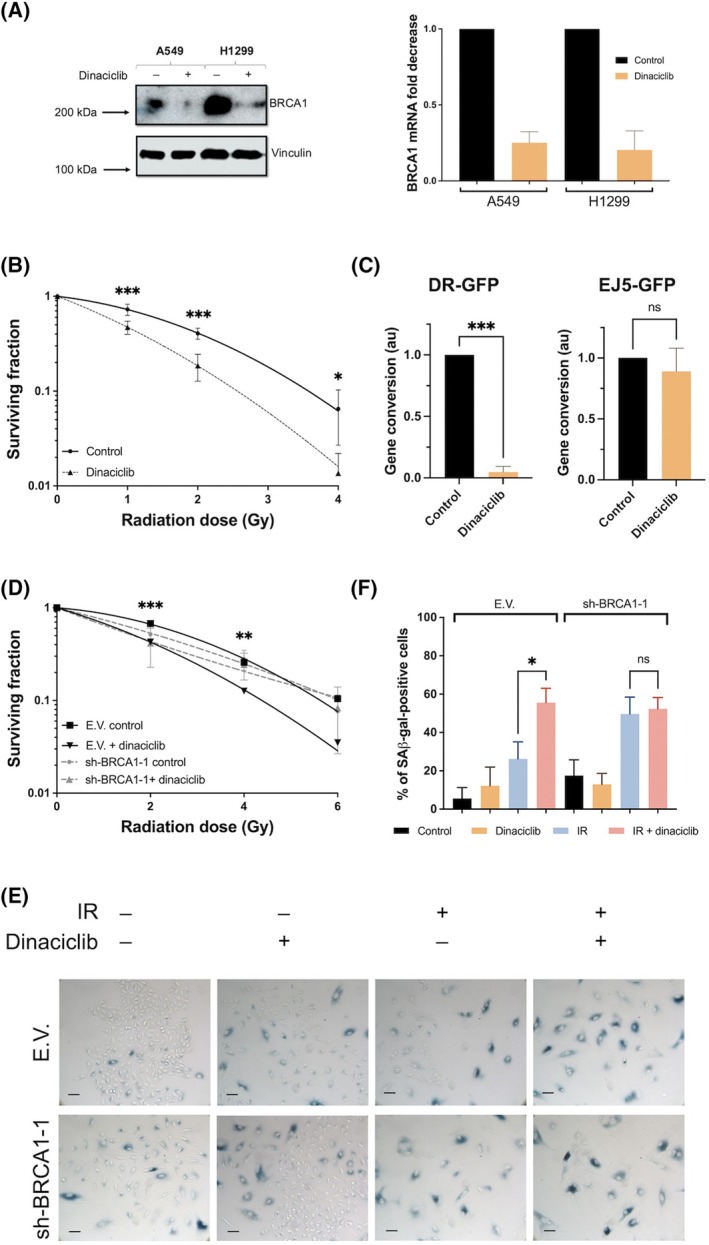
Downregulation of BRCA1 expression mediates radiosensitivity associated with dinaciclib. (A) BRCA1 expression was evaluated in A549 and H1299 cell lines after 24 h treatment of dinaciclib (10 nm) or vehicle by western blot using vinculin as a loading control (upper panel) and by RT‐qPCR (lower panel). Histogram showing the average of three independent experiments. Bars mean SD. (B) Clonogenic assays in U20S cells. Cells were plated and 24 h later exposed to vehicle or dinaciclib (10 nm) for additional 24 h. Then cells were exposed to the indicated doses of X rays and immediately after media was replaced. Surviving fraction was normalised to respective unirradiated controls. Curves were fitted using lineal‐quadratic model. Graphics represent the average of three independent experiment performed in triplicated. The two‐way ANOVA test was used to assess statistical significance. Bars mean SD. (C) GFP reporter assay in U20S using DR‐GFP (left histogram) or EJ5‐GFP (right histogram). Histogram showing the average of three independent experiments. The unpaired Student's *t*‐test was used to assess statistical significance. Bars mean SD. (D) A549 cells infected with lentiviruses carrying shRNA for BRCA1 (sh‐BRCA1‐1) or empty vector (E.V.) were exposed to the indicated doses of X‐rays in the presence of 24 h pretreatment of dinaciclib (10 nm) or vehicle. Cellular radiosensitivity was plotted using control cells. Graphics represent the average of three independent experiments (one per infection) performed in triplicated cultures. The two‐way ANOVA test was used to assess statistical significance. Bars mean SD. (E) A549 cells infected with lentiviruses carrying sh‐BRCA1 or empty vector (E.V.) were irradiated (6 Gy) in presence/absence of 24 h pretreatment of dinaciclib (10 nm) or vehicle and 6 days later SA‐β‐Gal activity was detected by X‐Gal staining. Images show a representative field out of five analysed per condition in three independent experiment. for SA‐β‐Gal stainin. Scale bars represent 100 μm. (F) Histogram showing the average of three independent experiments representing the percentage of positive cells. The unpaired Student's *t*‐test was used to assess statistical significance. Bars mean SD. The statistical significance of differences is indicated in figures by asterisks as follows: *P < 0.05, **P < 0.01 and ***P < 0.001. ns, not significative.

In the light of these findings, we next sought to investigate an upstream mechanism that controls the radiosensitive phenotype associated with dinaciclib. In this regard, abrogation of BRCA1 expression is one of the direct effects of dinaciclib [4]. As shown in Fig. 6A, a significant decrease in *BRCA1* expression, at both protein and RNA levels, was observed after exposure to dinaciclib in both models as well as in the other cell lines (Fig. S9). Indeed, another well‐established CDK inhibitor, palbociclib, which targets CDK4/6 in contrast to dinaciclib [29], showed no effect on *BRCA1* expression, reinforcing the specificity of our observations (Fig. S10). We therefore considered that the absence of BRCA1 could affect the DNA damage response and, more specifically to HR. In fact, we observed that A549 cells pretreated with dinaciclib showed a higher basal level of DNA damage, as judged by comet assays, although the recovery of damage in early time points was extremely similar to untreated cells, suggesting that the rapid DNA repair mechanism (e.g. NHEJ) seen to be unaffected by dinaciclib (Fig. S11).

To this end, we switched to an experimental model based on U2OS cells with functional p53 and HR [30]. As shown, dinaciclib was also able to promote radiosensitivity in U2OS cells (Fig. 6B). Furthermore, in agreement with a decrease in *BRCA1* expression, dinaciclib treatment showed a marked block of HR without affecting NHEJ, as measured by specific repair pathways reporters [24, 25] (Fig. 6C). To fully support the role of *BRCA1*, A549 cells were knocked down for BRCA1. After achieving effective knockdown (Fig. S12A), cells were exposed to IR in the presence/absence of dinaciclib pretreatment. As expected, the downregulation of *BRCA1* gene expression reduced the radiosensitizing potential associated with dinaciclib (Fig. 6D). In fact, in cells lacking *BRCA1* expression, a pronounced senescence phenotype was observed in response to IR that was unaffected by dinaciclib pretreatment, whereas in control cells, a marked increase in senescence was observed in dinaciclib‐pretreated cells after IR exposure compared with irradiated cells (Fig. 6E,F). Furthermore, all these results were confirmed by a second shRNA (Fig. S12B–D).

**Fig. 7:**
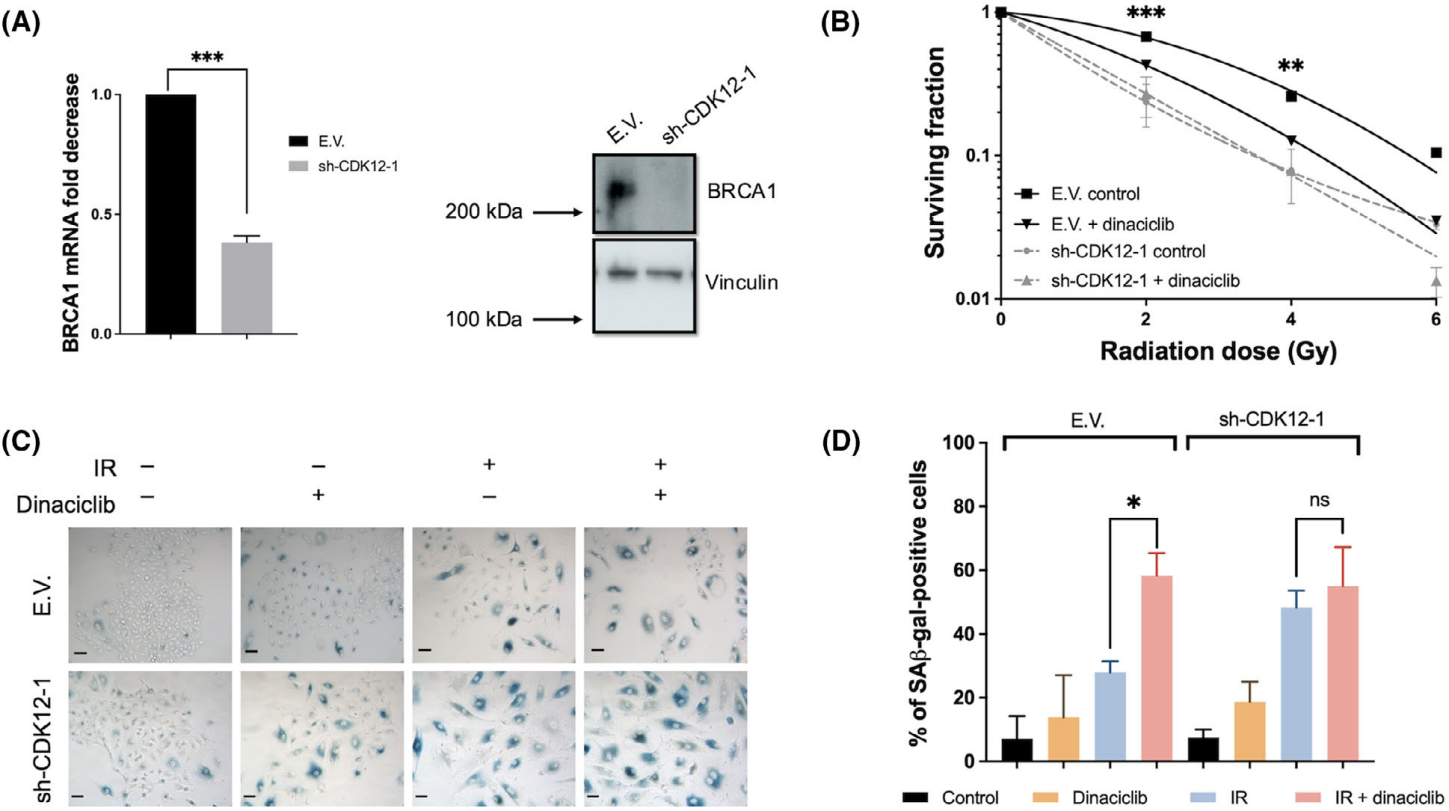
CDK12 inhibition is a key event dinaciclib‐associated radiosensitivity. (A) BRCA1 expression was evaluated in A549 cells infected with lentiviruses carrying shRNA for CDK12 (sh‐CDK12‐1). Selected pools were evaluated by RT‐qPCR (left panel average of three independent infections) and by western blot using tubulin as a loading control (right panel representative experiment out of 3). The unpaired Student's *t*‐test was used to assess statistical significance. Bars mean SD. (B) A549 cells infected with lentiviruses carrying sh‐CDK12‐1 or empty vector (E.V.) were exposed to the indicated doses of X‐rays in the presence of 24‐h pretreatment of dinaciclib (10 nm) or vehicle. Cellular radiosensitivity was plotted using control cells. Graphics represent the average of three independent experiment (one per infection) performed in triplicated cultures. The two‐way ANOVA test was used to assess statistical significance. Bars mean SD. (C) A549 cells infected with lentiviruses carrying sh‐CDK12‐1 or empty vector (E.V.) were irradiated (6 Gy) in presence of 24 h pretreatment of dinaciclib (10 nm) or vehicle and 6 days later SA‐β‐Gal activity was detected by X‐Gal staining. Images show a representative field out of five analysed per condition in three independent experiments. Scale bars represent 100 μm. (D) Histogram showing the average of three independent experiments representing the percentage of positive cells. The unpaired Student's *t*‐test was used to assess statistical significance. Bars mean SD. The statistical significance of differences is indicated in figures by asterisks as follows: *P < 0.05, **P < 0.01 and ***P < 0.001. ns, not significative.

Finally, we decided to investigate the role of *CDK12*, which has been proposed as a key mediator of dinaciclib blockade of *BRCA1* gene expression [4]. Therefore, silencing of *CDK12* expression was performed using shRNA (Fig. S13A). As shown, cells lacking *CDK12* expression showed a marked downregulation of BRCA1 expression at both RNA and protein level (Fig. 7A). To fully elucidate the role of this particular CDK in dinaciclib‐associated radiosensitivity, we performed a clonogenic assay in the presence/absence of dinaciclib pretreatment. Interestingly, dinaciclib showed a marked reduction in the induction of radiosensitivity in CDK12‐interfered cells but not in controls (Fig. 7B), which correlated with a marked senescent phenotype in interfered cells but again not in controls (Fig. 7C,D), showing exact behaviour that in the case of shRNA‐BRCA1 cells. To fully support these observations, a second shRNA against *CDK12* was challenged with almost identical results (Fig. S13B–D).

Therefore, these results support a model in which *CDK12* inhibition abrogates *BRCA1* expression, resulting in a nonfunctional HR that increases IR‐associated senescence in a *TP53*‐dependent manner directly implicated in dinaciclib‐associated radiosensitivity.

## 4 Discussion

Several conclusions are drawn from the present report.

First, dinaciclib is a novel and potent radiosensitiser that could be added to the growing list of cell cycle‐targeted therapies with the ability to induce radiosensitivity [31, 32], but it cannot be claimed to be a universal radiosensitiser. In this sense, our data allow the correct selection of patients who could benefit from a future combination therapy based on markers such as *BRCA1* or *TP53*, which are currently being evaluated in clinical practice. This observation could be of particular interest in pathologies such as breast or lung cancer, where radiotherapy is a cornerstone and dinaciclib is being considered for clinical use [6, 7, 33, 34].

Second, dinaciclib promotes radiosensitivity in a genetic context that requires at least functional *TP53* and *BRCA1*. Regarding *TP53*, our experimental data in several models (e.g., A549, HCT 116) support a definitive role for this tumour suppressor in the radiosensitivity associated with dinaciclib. In this regard, it is important to mention that *TP53* is one of the most common alterations in cancer present in approximately 50% of tumours [35], suggesting that our finding could be limited to a group of tumours with a theoretically better response to IR. However, several points highlight the importance of the current findings. First, the definitive role of *TP53* in cellular radioresistance is not clear [36]. Furthermore, no clinical protocol considers *TP53* status as a biomarker for radiotherapy. For example, *in silico* searches in The Cancer Genome Atlas Database (TCGA) lung squamous cell carcinoma cohort using the cBioPortal tool [37] showed that WT *TP53* tumours did not have a better prognosis than mutant *TP53* tumours, regardless of whether they were treated with radiotherapy or not (Fig. S14). Therefore, there is an urgent clinical need to optimise radiotherapy not only for *TP53* mutant tumours but also for WT *TP53* tumours, reinforcing the importance of our findings. Our observations on the role of *TP53*, obtained using different models such as H1299 or HT‐29 as well as shRNA experiments and HCT 116 null model showed results along the same lines as those obtained with palbociclib or abemaciclib [16, 38]. However, it is known that both CDK4/6 inhibitors directly affect ATM kinase activity and subsequent activation of p53 [16, 39], whereas dinaciclib had no effect on ATM signalling as assessed by H2AX or KAP phosphorylation. Notably, recent studies indicate that dinaciclib is able to inhibit ATM/CHK2 signalling in Hela and SiHa cells, suggesting that cell cycle and apoptosis are the main mechanisms of dinaciclib‐associated radiosensitivity [15]. However, despite the different experimental models used, these conclusions are based on a correlation that lacks genetic approaches and quantitative methods. In addition, it is noteworthy that in their two cellular models, IR is unable to activate CHK2 or H2AX phosphorylation in response to IR, which is totally opposite to our model as well as previous work in Hela cells showing normal activation of H2AX or CHK2 after IR [40, 41]. Furthermore, in terms of apoptosis, the results are similar to our study showing no synergistic effect, which clearly exclude apoptosis as a radiosensitizing mechanism. Finally, the cell cycle alteration shown by Zang and coworkers does not indicate whether dinaciclib is affecting G2/M exit after IR exposure and, interestingly, no increase in G2/M is observed after IR exposure in any of the cell lines, which is totally unexpected, and opposite to what we have shown in our experimental setting as well as previous works in Hela cells [42]. In conclusion, this previous work needs to be carefully re‐evaluated to avoid misinterpretation in order to optimise this novel combination.

Third, the effect of dinaciclib on *BRCA1* expression is a key issue, which seems to be specific for dinaciclib targets, as the use of palbociclib indicates (Fig. S10). Second, it has been shown that cells lacking BRCA1 are extremely sensitive to IR [43] and prone to senescence [44] mediated by *TP53* [45], fitting perfectly with our proposed model of dinaciclib‐associated radiosensitivity. In this sense, dinaciclib could be acting in a similar way to other HR blockers as tanespimycin [46], a known inhibitor of Hsp90 that blocks HR [47] and confer radiosensitivity by increasing *TP53*‐dependent senescence through its effect on BRCA1 proteasomal degradation [48, 49]. Furthermore, in an attempt to bring our observation to the clinical level, we performed *in silico* studies (Fig. S15) about the level of *BRCA1* expression in the TCGA lung squamous cell carcinoma cohort. Interestingly, we did not observe correlation with overall survival (*P*‐value: 0.487). However, if we analyse the population that received radiotherapy, 52 of 486 patients, we observe that *BRCA1* expression levels almost reach statistical significance (Fig. S15B, *P*‐value: 0.0532), suggesting that high levels of BRCA1 may mediate a poor response to radiotherapy. Although these data may support our hypothesis, further studies are needed to draw more definitive conclusions at the clinical level as has been proposed for BRCA1 mutations [50]. However, other genes that are also regulated by dinaciclib should be taken into account. This could be the case for *RAD51*, which is regulated by dinaciclib [4] and is implicated in the HR process [51], and is known to control cellular radiosensitivity [52, 53].

In addition, our data suggest that senescence is the critical biological process for the radiosensitizing effect associated with dinaciclib, providing the first link between radiation‐induced senescence and dinaciclib. This observation opens the possibility of considering senolytics as a new player in our proposed chemo/radiotherapy regimen. However, it is important to note that the prosenescent effect of dinaciclib could be stimulus‐dependent, as has been shown in the case of doxorubicin [11] but not for arginine‐deprivation [54]. Therefore, our data support the possibility of a therapeutic regimen based on dinaciclib/radiotherapy/senolytics in a sequential manner, which could significantly improve the potential of radiotherapy [55, 56]. Furthermore, our observation may have implications in others therapeutic approaches as immunotherapy in which senescence has recently been shown to be involved [57] and interestingly could explain the effect observed for the combination of dinaciclib and immunotherapy based on anti‐PD‐1 [58]. In this sense, our observations could have potential implication in pathologies such as multiple myeloma or sarcoma in which the combination of senolytics and dinaciclib have shown interesting results [59, 60].

Finally, another interesting question raised by our data is the role of CDK12 in the combination of dinaciclib and radiotherapy. Our data support the role of CDK12 activity onto *BRCA1* expression as a one of the possible effectors in the role of dinaciclib as a radiosensitizing agent. However, we should consider that dinaciclib affect several molecules implicated in HR as has been demonstrated in breast cancer [4] that could be critical in the induction of radiosensitivity mediated by dinaciclib and need to be further evaluated. In addition, we cannot rule out other dinaciclib targets as CDK9, which has been reported to control BRCA1 recruitment to DNA damage sites [61] and also expression levels [62]. Finally, our findings open the possibility to consider CDK12 inhibitors not only as new targeted therapy agents [63, 64] but also as potent radiosensitizing agents trough the modulation of radiation‐induced senescence.

## 5 Conclusion

In conclusion, we present new evidence that dinaciclib is a potent radiosensitizing agent through its inhibitory effect on CDK12, which downregulates the expression of key components of the HR machinery such as *BRCA1*. This event promotes a senescent phenotype that requires a WT *TP53*. Our observations provide the rationale for a future combination of radiotherapy plus dinaciclib in those patients with at least a functional BRCA1/p53 axis, which accounts for approximately 50% of cases, allowing for more selective and effective radiotherapy.

## Conflicts of interest

The authors declare no conflict of interest.

## Author contributions

NGF, DMF‐A, CG‐G, AD‐C, JJ‐S, PF‐A, FJC and BB performed the experimental procedures, and participated in the data analysis. SS, and IA supervised the irradiation procedures. BB, GC, IP, PH and MJR‐H provided intellectual input, participate in the data analysis and correct the manuscript. RS‐P conceived and supervised the study, gets funding and wrote the paper with the help of NGF.

### Peer review

The peer review history for this article is available at https://www.webofscience.com/api/gateway/wos/peer‐review/10.1002/1878‐0261.13773.

## Supporting information


**Fig. S1.** Dose–response assay to dinaciclib in the different cell lines used.

**Fig. S2.** Cell cycle profiles in A549 cells.

**Fig. S3.** Evaluation of p53 and p21 expression in response to ionising radiation in the different cell lines used.

**Fig. S4.** Apoptosis assays in HCT 116 and HT‐29 cell lines.

**Fig. S5.** SA‐β‐Gal activity detection in HCT 116 and HT‐29 cell lines.

**Fig. S6.** Evaluation of p21 expression in A549 and H1299 cell lines 6 days after ionising radiation exposure.

**Fig. S7.** SA‐β‐Gal activity detection in the presence/absence of navitoclax treatment.

**Fig. S8.** Study of dinaciclib effects in the isogenic c model of HCT 116 (HCT 116 p53 +/+ and −/).

**Fig. S9.** Effect of dinaciclib onto BRCA1 expression in HCT 116 and HT‐29 cell lines.

**Fig. S10.** Effect of palbociclib onto BRCA1 expression in A549 cell line.

**Fig. S11.** Comet assay in A549 cells with or without dinaciclib pretreatment.

**Fig. S12.** Effect of BRCA1 interference (sh‐BRCA1‐2) on dinaciclib‐associated radiosensitivity in the A549 cell line.

**Fig. S13.** Effect of CDK12 interference (sh‐CDK12‐2) on dinaciclib‐associated radiosensitivity in the A549 cell line.

**Fig. S14.** *In silico* study of *TP53* mutation in the overall survival of the lung squamous cell carcinoma cohort from TCGA.

**Fig. S15.** *In silico* study of *BRCA1* expression (mRNA) in the overall survival of the lung squamous cell carcinoma cohort from TCGA.

**Table S1.** List of antibodies used.

**Table S2.** List of primers used.

## Data Availability

The data that support the findings of this study are available from the corresponding author (rsprieto@iib.uam.es) upon reasonable request.

## References

[1] Schettini F , Giudici F , Giuliano M , Cristofanilli M , Arpino G , Del Mastro L , et al. Overall survival of CDK4/6‐inhibitor‐based treatments in clinically relevant subgroups of metastatic breast cancer: systematic review and meta‐analysis. J Natl Cancer Inst. 2020;112(11):1089–1097. 32407488 10.1093/jnci/djaa071 PMC7669227

[2] Lelliott EJ , Sheppard KE , McArthur GA . Harnessing the immunotherapeutic potential of CDK4/6 inhibitors in melanoma: is timing everything? NPJ Precis Oncol. 2022;6(1):26. 35444175 10.1038/s41698-022-00273-9 PMC9021218

[3] Parry D , Guzi T , Shanahan F , Davis N , Prabhavalkar D , Wiswell D , et al. Dinaciclib (SCH 727965), a novel and potent cyclin‐dependent kinase inhibitor. Mol Cancer Ther. 2010;9(8):2344–2353. 20663931 10.1158/1535-7163.MCT-10-0324

[4] Johnson SF , Cruz C , Greifenberg AK , Dust S , Stover DG , Chi D , et al. CDK12 inhibition reverses de novo and acquired PARP inhibitor resistance in BRCA wild‐type and mutated models of triple‐negative breast cancer. Cell Rep. 2016;17(9):2367–2381. 27880910 10.1016/j.celrep.2016.10.077 PMC5176643

[5] Paculová H , Kohoutek J . The emerging roles of CDK12 in tumorigenesis. Cell Div. 2017;12:7. 29090014 10.1186/s13008-017-0033-x PMC5658942

[6] Gregory GP , Kumar S , Wang D , Mahadevan D , Walker P , Wagner‐Johnston N , et al. Pembrolizumab plus dinaciclib in patients with hematologic malignancies: the phase 1b KEYNOTE‐155 study. Blood Adv. 2022;6(4):1232–1242. 34972202 10.1182/bloodadvances.2021005872 PMC8864641

[7] Murphy AG , Zahurak M , Shah M , Weekes CD , Hansen A , Siu LL , et al. A phase I study of dinaciclib in combination with MK‐2206 in patients with advanced pancreatic cancer. Clin Transl Sci. 2020;13(6):1178–1188. 32738099 10.1111/cts.12802 PMC7719383

[8] Fu W , Ma L , Chu B , Wang X , Bui MM , Gemmer J , et al. The cyclin‐dependent kinase inhibitor SCH 727965 (dinacliclib) induces the apoptosis of osteosarcoma cells. Mol Cancer Ther. 2011;10(6):1018–1027. 21490307 10.1158/1535-7163.MCT-11-0167 PMC4727401

[9] Saqub H , Proetsch‐Gugerbauer H , Bezrookove V , Nosrati M , Vaquero EM , de Semir D , et al. Dinaciclib, a cyclin‐dependent kinase inhibitor, suppresses cholangiocarcinoma growth by targeting CDK2/5/9. Sci Rep. 2020;10(1):18489. 33116269 10.1038/s41598-020-75578-5 PMC7595101

[10] Zhu Y , Liu Y , Zhang C , Chu J , Wu Y , Li Y , et al. Tamoxifen‐resistant breast cancer cells are resistant to DNA‐damaging chemotherapy because of upregulated BARD1 and BRCA1. Nat Commun. 2018;9(1):1595. 29686231 10.1038/s41467-018-03951-0 PMC5913295

[11] Tang H , Xu L , Liang X , Gao G . Low dose dinaciclib enhances doxorubicin‐induced senescence in myeloma RPMI8226 cells by transformation of the p21 and p16 pathways. Oncol Lett. 2018;16(5):6608–6614. 30405800 10.3892/ol.2018.9474 PMC6202540

[12] Chen X‐X , Xie F‐F , Zhu X‐J , Lin F , Pan S‐S , Gong L‐H , et al. Cyclin‐dependent kinase inhibitor dinaciclib potently synergizes with cisplatin in preclinical models of ovarian cancer. Oncotarget. 2015;6(17):14926–14939. 25962959 10.18632/oncotarget.3717 PMC4558126

[13] Jane EP , Premkumar DR , Cavaleri JM , Sutera PA , Rajasekar T , Pollack IF . Dinaciclib, a cyclin‐dependent kinase inhibitor promotes proteasomal degradation of mcl‐1 and enhances ABT‐737–mediated cell death in malignant human glioma cell lines. J Pharmacol Exp Ther. 2016;356(2):354–365. 26585571 10.1124/jpet.115.230052 PMC6047232

[14] Carey JPW , Karakas C , Bui T , Chen X , Vijayaraghavan S , Zhao Y , et al. Synthetic lethality of PARP inhibitors in combination with MYC blockade is independent of BRCA status in triple‐negative breast cancer. Cancer Res. 2018;78(3):742–757. 29180466 10.1158/0008-5472.CAN-17-1494 PMC5811386

[15] Zhang H , Chu T , Zheng J , Teng Y , Ma R , Zou L , et al. Sensitization of cervical cancer cells to radiation by the cyclin‐dependent kinase inhibitor dinaciclib. Med Oncol. 2022;40(2):68. 36586018 10.1007/s12032-022-01890-x

[16] Fernández‐Aroca DM , Roche O , Sabater S , Pascual‐Serra R , Ortega‐Muelas M , Sánchez Pérez I , et al. P53 pathway is a major determinant in the radiosensitizing effect of palbociclib: implication in cancer therapy. Cancer Lett. 2019;451:23–33. 30872077 10.1016/j.canlet.2019.02.049

[17] Pascual‐Serra R , Fernández‐Aroca DM , Sabater S , Roche O , Andrés I , Ortega‐Muelas M , et al. p38β (MAPK11) mediates gemcitabine‐associated radiosensitivity in sarcoma experimental models. Radiother Oncol. 2021;156:136–144. 33310004 10.1016/j.radonc.2020.12.008

[18] Valero ML , Cimas FJ , Arias L , Melgar‐Rojas P , García E , Callejas‐Valera JL , et al. E1a promotes c‐Myc‐dependent replicative stress: implications in glioblastoma radiosensitization. Cell Cycle. 2014;13(1):52–61. 24196438 10.4161/cc.26754 PMC3925735

[19] González JE , Lee M , Barquinero JF , Valente M , Roch‐Lefèvre S , García O . Quantitative image analysis of gamma‐H2AX foci induced by ionizing radiation applying open source programs. Anal Quant Cytol Histol. 2012;34(2):66–71. 22611761

[20] de la Cruz‐Morcillo MA , García‐Cano J , Arias‐González L , García‐Gil E , Artacho‐Cordón F , Ríos‐Arrabal S , et al. Abrogation of the p38 MAPK α signaling pathway does not promote radioresistance but its activity is required for 5‐fluorouracil‐associated radiosensitivity. Cancer Lett. 2013;335(1):66–74. 23403078 10.1016/j.canlet.2013.01.050

[21] Franken NAP , Rodermond HM , Stap J , Haveman J , van Bree C . Clonogenic assay of cells in vitro. Nat Protoc. 2006;1(5):2315–2319. 17406473 10.1038/nprot.2006.339

[22] van Leeuwen CM , Oei AL , Crezee J , Bel A , Franken NAP , Stalpers LJA , et al. The alfa and beta of tumours: a review of parameters of the linear‐quadratic model, derived from clinical radiotherapy studies. Radiat Oncol. 2018;13(1):96. 29769103 10.1186/s13014-018-1040-z PMC5956964

[23] Adrados I , Larrasa‐Alonso J , Galarreta A , López‐Antona I , Menéndez C , Abad M , et al. The homeoprotein SIX1 controls cellular senescence through the regulation of p16INK4A and differentiation‐related genes. Oncogene. 2016;35(27):3485–3494. 26500063 10.1038/onc.2015.408 PMC5730042

[24] Pierce AJ , Johnson RD , Thompson LH , Jasin M . XRCC3 promotes homology‐directed repair of DNA damage in mammalian cells. Genes Dev. 1999;13(20):2633–2638. 10541549 10.1101/gad.13.20.2633 PMC317094

[25] Bennardo N , Cheng A , Huang N , Stark JM . Alternative‐NHEJ is a mechanistically distinct pathway of mammalian chromosome break repair. PLoS Genet. 2008;4(6):e1000110. 18584027 10.1371/journal.pgen.1000110 PMC2430616

[26] Rodríguez‐Real G , Domínguez‐Calvo A , Prados‐Carvajal R , Bayona‐Feliú A , Gomes‐Pereira S , Balestra FR , et al. Centriolar subdistal appendages promote double‐strand break repair through homologous recombination. EMBO Rep. 2023;24(10):e56724. 37664992 10.15252/embr.202256724 PMC10561181

[27] Leroy B , Girard L , Hollestelle A , Minna JD , Gazdar AF , Soussi T . Analysis of TP53 mutation status in human cancer cell lines: a reassessment. Hum Mutat. 2014;35(6):756–765. 24700732 10.1002/humu.22556 PMC4451114

[28] Wissler Gerdes EO , Zhu Y , Tchkonia T , Kirkland JL . Discovery, development, and future application of senolytics: theories and predictions. FEBS J. 2020;287(12):2418–2427. 32112672 10.1111/febs.15264 PMC7302972

[29] Fry DW , Harvey PJ , Keller PR , Elliott WL , Meade M , Trachet E , et al. Specific inhibition of cyclin‐dependent kinase 4/6 by PD 0332991 and associated antitumor activity in human tumor xenografts. Mol Cancer Ther. 2004;3(11):1427–1438. 15542782

[30] Bian J , Sun Y . Transcriptional activation by p53 of the human type IV collagenase (gelatinase A or matrix metalloproteinase 2) promoter. Mol Cell Biol. 1997;17(11):6330–6338. 9343394 10.1128/mcb.17.11.6330 PMC232484

[31] Hauge S , Eek Mariampillai A , Rødland GE , Bay LTE , Landsverk HB , Syljuåsen RG . Expanding roles of cell cycle checkpoint inhibitors in radiation oncology. Int J Radiat Biol. 2023;99(6):941–950. 33877959 10.1080/09553002.2021.1913529

[32] Yang Y , Luo J , Chen X , Yang Z , Mei X , Ma J , et al. CDK4/6 inhibitors: a novel strategy for tumor radiosensitization. J Exp Clin Cancer Res. 2020;39(1):188. 32933570 10.1186/s13046-020-01693-w PMC7490904

[33] Mitri Z , Karakas C , Wei C , Briones B , Simmons H , Ibrahim N , et al. A phase 1 study with dose expansion of the CDK inhibitor dinaciclib (SCH 727965) in combination with epirubicin in patients with metastatic triple negative breast cancer. Invest New Drugs. 2015;33(4):890–894. 25947565 10.1007/s10637-015-0244-4

[34] Stephenson JJ , Nemunaitis J , Joy AA , Martin JC , Jou Y‐M , Zhang D , et al. Randomized phase 2 study of the cyclin‐dependent kinase inhibitor dinaciclib (MK‐7965) versus erlotinib in patients with non‐small cell lung cancer. Lung Cancer. 2014;83(2):219–223. 24388167 10.1016/j.lungcan.2013.11.020

[35] Levine AJ . p53: 800 million years of evolution and 40 years of discovery. Nat Rev Cancer. 2020;20(8):471–480. 32404993 10.1038/s41568-020-0262-1

[36] Slichenmyer WJ , Nelson WG , Slebos RJ , Kastan MB . Loss of a p53‐associated G1 checkpoint does not decrease cell survival following DNA damage. Cancer Res. 1993;53(18):4164–4168. 8364909

[37] Cerami E , Gao J , Dogrusoz U , Gross BE , Sumer SO , Aksoy BA , et al. The cBio cancer genomics portal: an open platform for exploring multidimensional cancer genomics data. Cancer Discov. 2012;2(5):401–404. 22588877 10.1158/2159-8290.CD-12-0095 PMC3956037

[38] Naz S , Sowers A , Choudhuri R , Wissler M , Gamson J , Mathias A , et al. Abemaciclib, a selective CDK4/6 inhibitor, enhances the radiosensitivity of non‐small cell lung cancer in vitro and in vivo. Clin Cancer Res. 2018;24(16):3994–4005. 29716919 10.1158/1078-0432.CCR-17-3575 PMC6137329

[39] Huang C‐Y , Hsieh F‐S , Wang C‐Y , Chen L‐J , Chang S‐S , Tsai M‐H , et al. Palbociclib enhances radiosensitivity of hepatocellular carcinoma and cholangiocarcinoma via inhibiting ataxia telangiectasia‐mutated kinase‐mediated DNA damage response. Eur J Cancer. 2018;102:10–22. 30103095 10.1016/j.ejca.2018.07.010

[40] Shao C‐S , Feng N , Zhou S , Zheng X‐X , Wang P , Zhang J‐S , et al. Ganoderic acid T improves the radiosensitivity of HeLa cells via converting apoptosis to necroptosis. Toxicol Res (Camb). 2021;10(3):531–541. 34141167 10.1093/toxres/tfab030 PMC8201584

[41] Yu Z‐J , Luo H‐H , Shang Z‐F , Guan H , Xiao B‐B , Liu X‐D , et al. Stabilization of 4E‐BP1 by PI3K kinase and its involvement in CHK2 phosphorylation in the cellular response to radiation. Int J Med Sci. 2017;14(5):452–461. 28539821 10.7150/ijms.18329 PMC5441037

[42] Cui F , Hou J , Huang C , Sun X , Zeng Y , Cheng H , et al. C‐Myc regulates radiation‐induced G2/M cell cycle arrest and cell death in human cervical cancer cells. J Obstet Gynaecol Res. 2017;43(4):729–735. 28150398 10.1111/jog.13261

[43] Shen SX , Weaver Z , Xu X , Li C , Weinstein M , Chen L , et al. A targeted disruption of the murine Brca1 gene causes gamma‐irradiation hypersensitivity and genetic instability. Oncogene. 1998;17(24):3115–3124. 9872327 10.1038/sj.onc.1202243

[44] Sedic M , Skibinski A , Brown N , Gallardo M , Mulligan P , Martinez P , et al. Haploinsufficiency for BRCA1 leads to cell‐type‐specific genomic instability and premature senescence. Nat Commun. 2015;6:7505. 26106036 10.1038/ncomms8505 PMC4491827

[45] Cao L , Li W , Kim S , Brodie SG , Deng C‐X . Senescence, aging, and malignant transformation mediated by p53 in mice lacking the Brca1 full‐length isoform. Genes Dev. 2003;17(2):201–213. 12533509 10.1101/gad.1050003 PMC195980

[46] Schulte TW , Neckers LM . The benzoquinone ansamycin 17‐allylamino‐17‐demethoxygeldanamycin binds to HSP90 and shares important biologic activities with geldanamycin. Cancer Chemother Pharmacol. 1998;42(4):273–279. 9744771 10.1007/s002800050817

[47] Noguchi M , Yu D , Hirayama R , Ninomiya Y , Sekine E , Kubota N , et al. Inhibition of homologous recombination repair in irradiated tumor cells pretreated with Hsp90 inhibitor 17‐allylamino‐17‐demethoxygeldanamycin. Biochem Biophys Res Commun. 2006;351(3):658–663. 17083915 10.1016/j.bbrc.2006.10.094

[48] Stecklein SR , Kumaraswamy E , Behbod F , Wang W , Chaguturu V , Harlan‐Williams LM , et al. BRCA1 and HSP90 cooperate in homologous and non‐homologous DNA double‐strand‐break repair and G2/M checkpoint activation. Proc Natl Acad Sci USA. 2012;109(34):13650–13655. 22869732 10.1073/pnas.1203326109 PMC3427093

[49] Shintani S , Zhang T , Aslam A , Sebastian K , Yoshimura T , Hamakawa H . P53‐dependent radiosensitizing effects of Hsp90 inhibitor 17‐allylamino‐17‐demethoxygeldanamycin on human oral squamous cell carcinoma cell lines. Int J Oncol. 2006;29(5):1111–1117. 17016641 10.3892/ijo.29.5.1111

[50] Kan C , Zhang J . BRCA1 mutation: a predictive marker for radiation therapy? Int J Radiat Oncol Biol Phys. 2015;93(2):281–293. 26383678 10.1016/j.ijrobp.2015.05.037 PMC4576355

[51] Demeyer A , Benhelli‐Mokrani H , Chénais B , Weigel P , Fleury F . Inhibiting homologous recombination by targeting RAD51 protein. Biochim Biophys Acta Rev Cancer. 2021;1876(2):188597. 34332021 10.1016/j.bbcan.2021.188597

[52] Taki T , Ohnishi T , Yamamoto A , Hiraga S , Arita N , Izumoto S , et al. Antisense inhibition of the RAD51 enhances radiosensitivity. Biochem Biophys Res Commun. 1996;223(2):434–438. 8670299 10.1006/bbrc.1996.0911

[53] Russell JS , Brady K , Burgan WE , Cerra MA , Oswald KA , Camphausen K , et al. Gleevec‐mediated inhibition of Rad51 expression and enhancement of tumor cell radiosensitivity. Cancer Res. 2003;63(21):7377–7383. 14612536

[54] Riess C , Del Moral K , Fiebig A , Kaps P , Linke C , Hinz B , et al. Implementation of a combined CDK inhibition and arginine‐deprivation approach to target arginine‐auxotrophic glioblastoma multiforme cells. Cell Death Dis. 2022;13(6):555. 35717443 10.1038/s41419-022-05006-1 PMC9206658

[55] Kim JH , Brown SL , Gordon MN . Radiation‐induced senescence: therapeutic opportunities. Radiat Oncol. 2023;18(1):10. 36639774 10.1186/s13014-022-02184-2 PMC9837958

[56] Bousset L , Gil J . Targeting senescence as an anticancer therapy. Mol Oncol. 2022;16(21):3855–3880. 36065138 10.1002/1878-0261.13312 PMC9627790

[57] Marin I , Boix O , Garcia‐Garijo A , Sirois I , Caballe A , Zarzuela E , et al. Cellular senescence is immunogenic and promotes antitumor immunity. Cancer Discov. 2023;13(2):410–431. 36302218 10.1158/2159-8290.CD-22-0523 PMC7614152

[58] Hossain DMS , Javaid S , Cai M , Zhang C , Sawant A , Hinton M , et al. Dinaciclib induces immunogenic cell death and enhances anti‐PD1–mediated tumor suppression. J Clin Invest. 2018;128(2):644–654. 29337311 10.1172/JCI94586 PMC5785250

[59] Beltrán‐Visiedo M , Jiménez‐Alduán N , Díez R , Cuenca M , Benedi A , Serrano‐Del Valle A , et al. Dinaciclib synergizes with BH3 mimetics targeting BCL‐2 and BCL‐XL in multiple myeloma cell lines partially dependent on MCL‐1 and in plasma cells from patients. Mol Oncol. 2023;17(12):2507–2525. 37704591 10.1002/1878-0261.13522 PMC10701777

[60] Rello‐Varona S , Fuentes‐Guirado M , López‐Alemany R , Contreras‐Pérez A , Mulet‐Margalef N , García‐Monclús S , et al. Bcl‐xL inhibition enhances dinaciclib‐induced cell death in soft‐tissue sarcomas. Sci Rep. 2019;9(1):3816. 30846724 10.1038/s41598-019-40106-7 PMC6405759

[61] Nepomuceno TC , Fernandes VC , Gomes TT , Carvalho RS , Suarez‐Kurtz G , Monteiro AN , et al. BRCA1 recruitment to damaged DNA sites is dependent on CDK9. Cell Cycle. 2017;16(7):665–672. 28278048 10.1080/15384101.2017.1295177 PMC5397266

[62] Li J , Zhi X , Chen S , Shen X , Chen C , Yuan L , et al. CDK9 inhibitor CDKI‐73 is synergetic lethal with PARP inhibitor olaparib in BRCA1 wide‐type ovarian cancer. Am J Cancer Res. 2020;10(4):1140–1155. 32368391 PMC7191097

[63] Xiao L , Liu Y , Chen H , Shen L . Targeting CDK9 with selective inhibitors or degraders in tumor therapy: an overview of recent developments. Cancer Biol Ther. 2023;24(1):2219470. 37272701 10.1080/15384047.2023.2219470 PMC10243401

[64] Yan Z , Du Y , Zhang H , Zheng Y , Lv H , Dong N , et al. Research progress of anticancer drugs targeting CDK12. RSC Med Chem. 2023;14(9):1629–1644. 37731700 10.1039/d3md00004d PMC10507796

