# Supplementary figures and images for: The CDK12–BRCA1 signaling axis mediates dinaciclib‐associated radiosensitivity through p53‐mediated cellular senescence
Source: Mol Oncol. 2024 Dec 3;19(4):1265–80. doi: 10.1002/1878-0261.13773 (PMC11977655; doi:10.1002/1878-0261.13773)

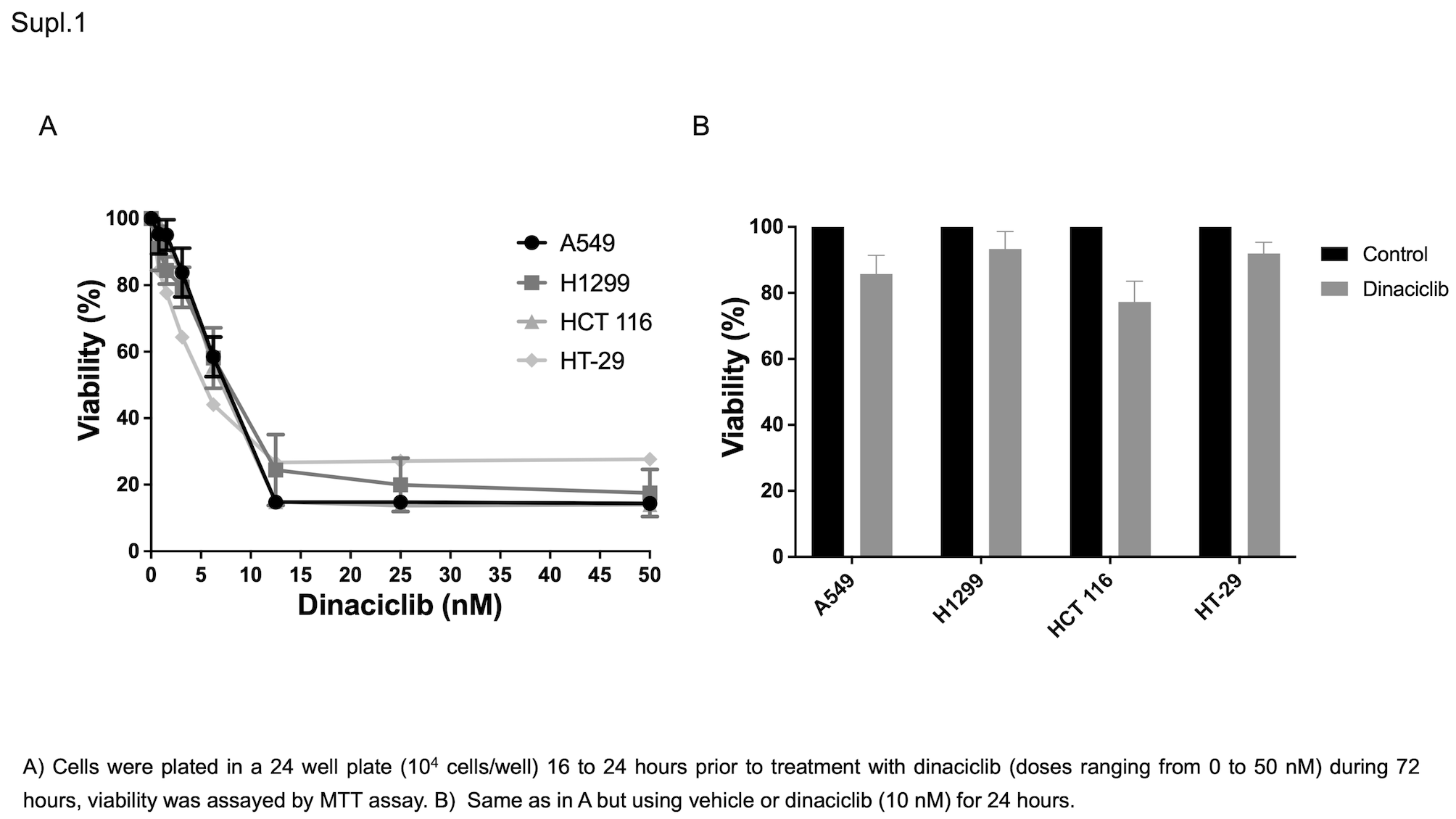

Supplement: Supplementary file 1 — Fig. S1. Dose–response assay to dinaciclib in the different cell lines used. [file MOL2-19-1265-s009.tiff]

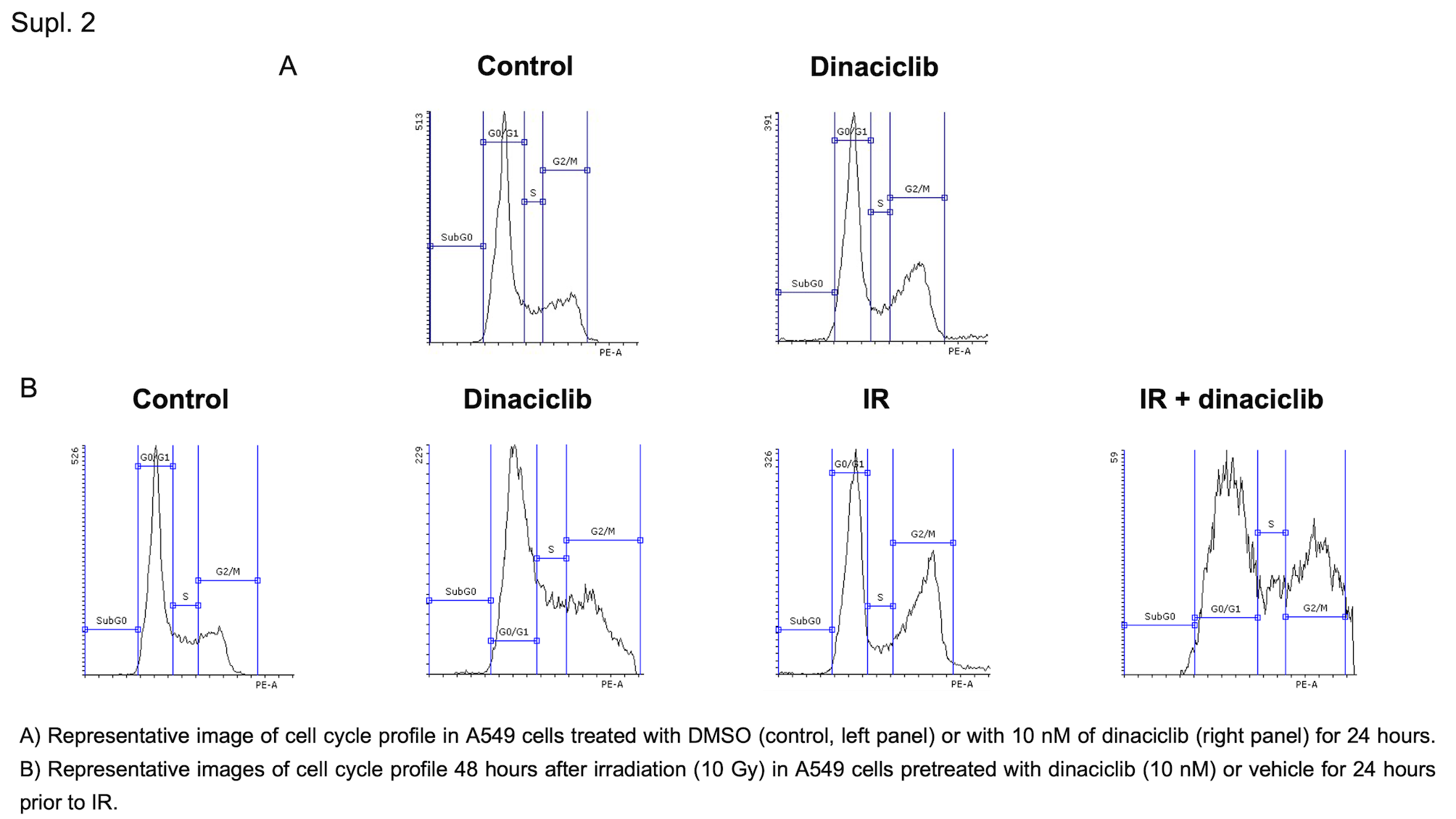

Supplement: Supplementary file 2 — Fig. S2. Cell cycle profiles in A549 cells. [file MOL2-19-1265-s002.tiff]

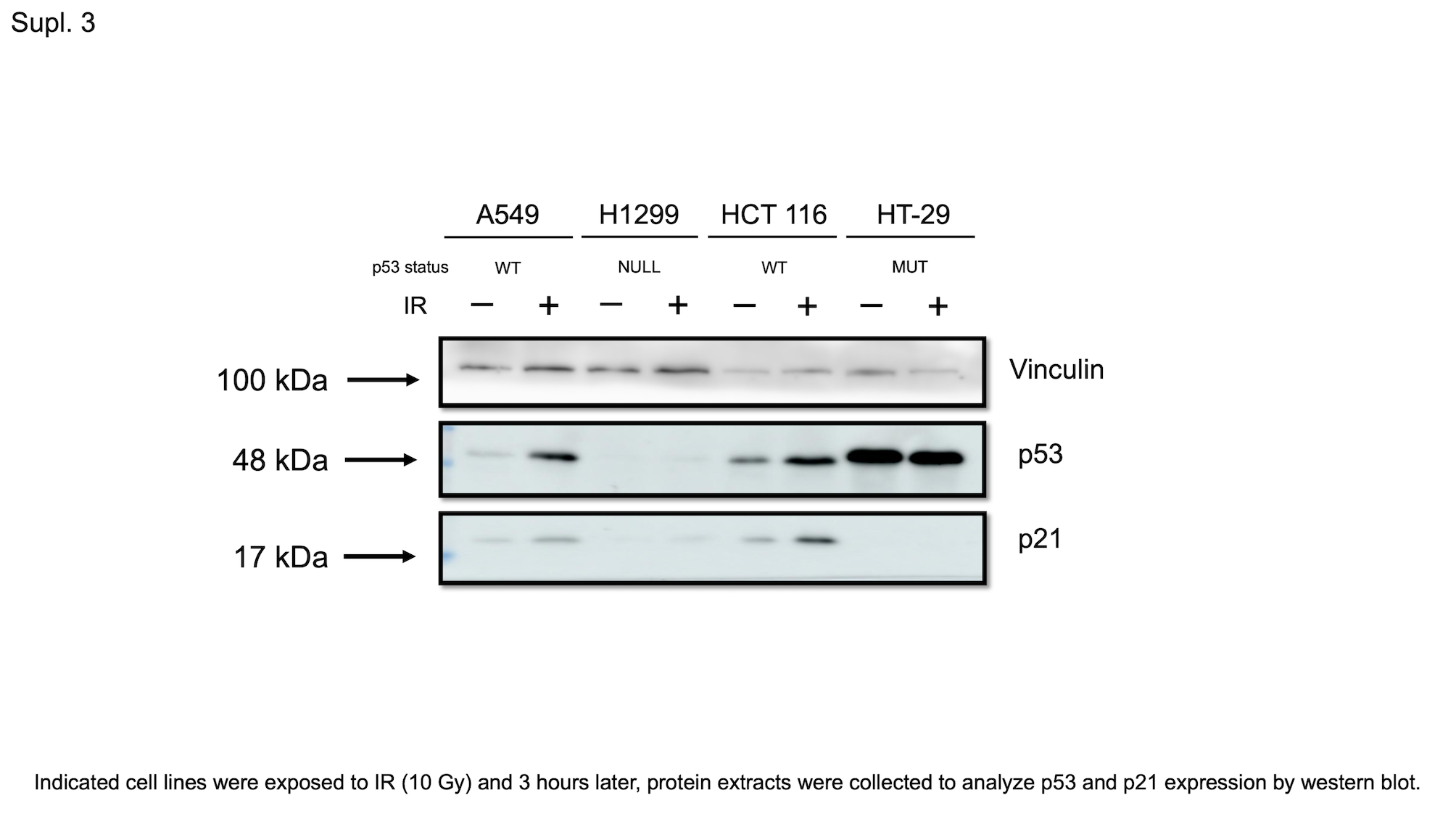

Supplement: Supplementary file 3 — Fig. S3. Evaluation of p53 and p21 expression in response to ionising radiation in the different cell lines used. [file MOL2-19-1265-s006.tiff]

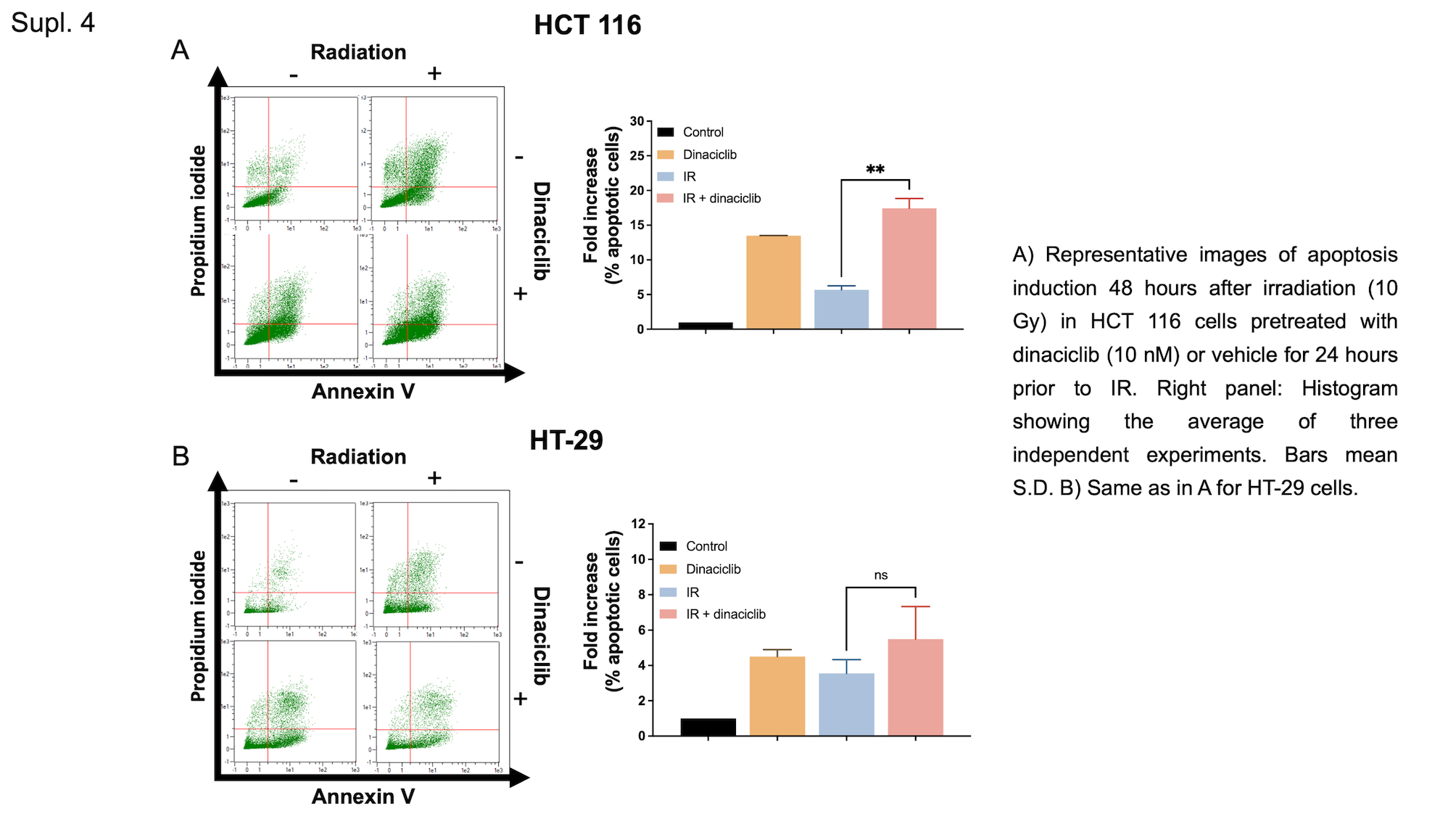

Supplement: Supplementary file 4 — Fig. S4. Apoptosis assays in HCT 116 and HT‐29 cell lines. [file MOL2-19-1265-s014.tiff]

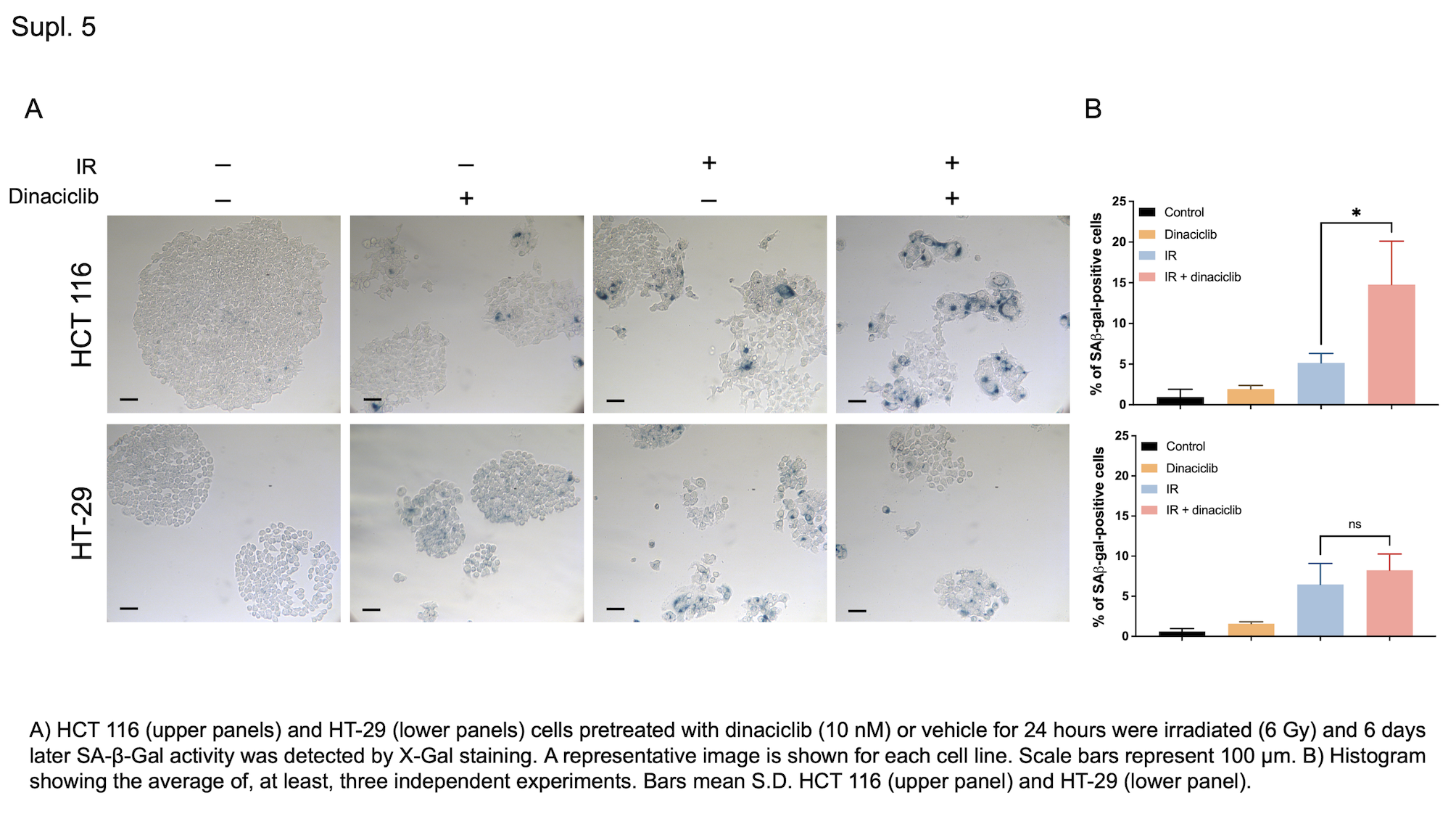

Supplement: Supplementary file 5 — Fig. S5. SA‐β‐Gal activity detection in HCT 116 and HT‐29 cell lines. [file MOL2-19-1265-s005.tiff]

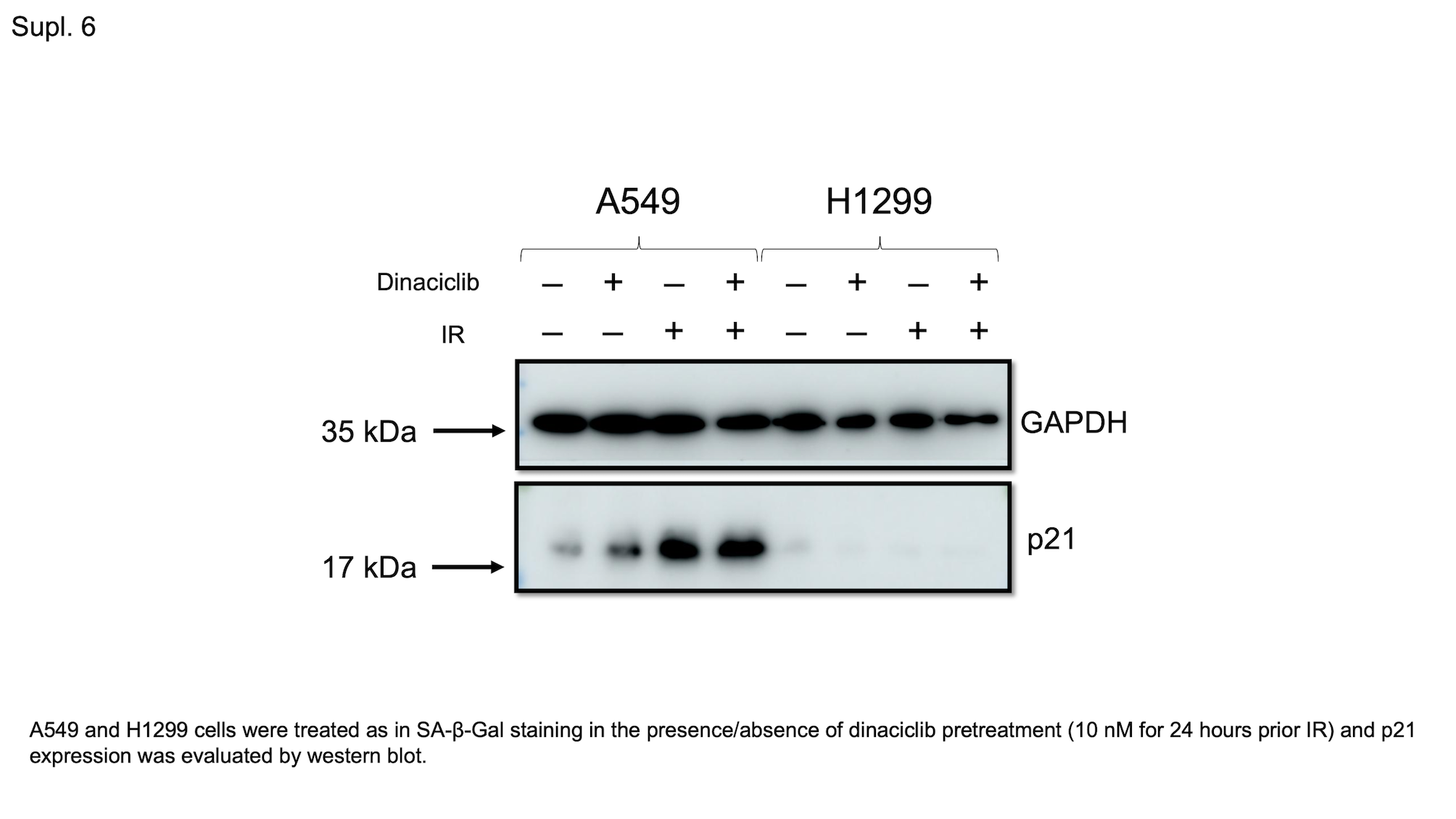

Supplement: Supplementary file 6 — Fig. S6. Evaluation of p21 expression in A549 and H1299 cell lines 6 days after ionising radiation exposure. [file MOL2-19-1265-s008.tiff]

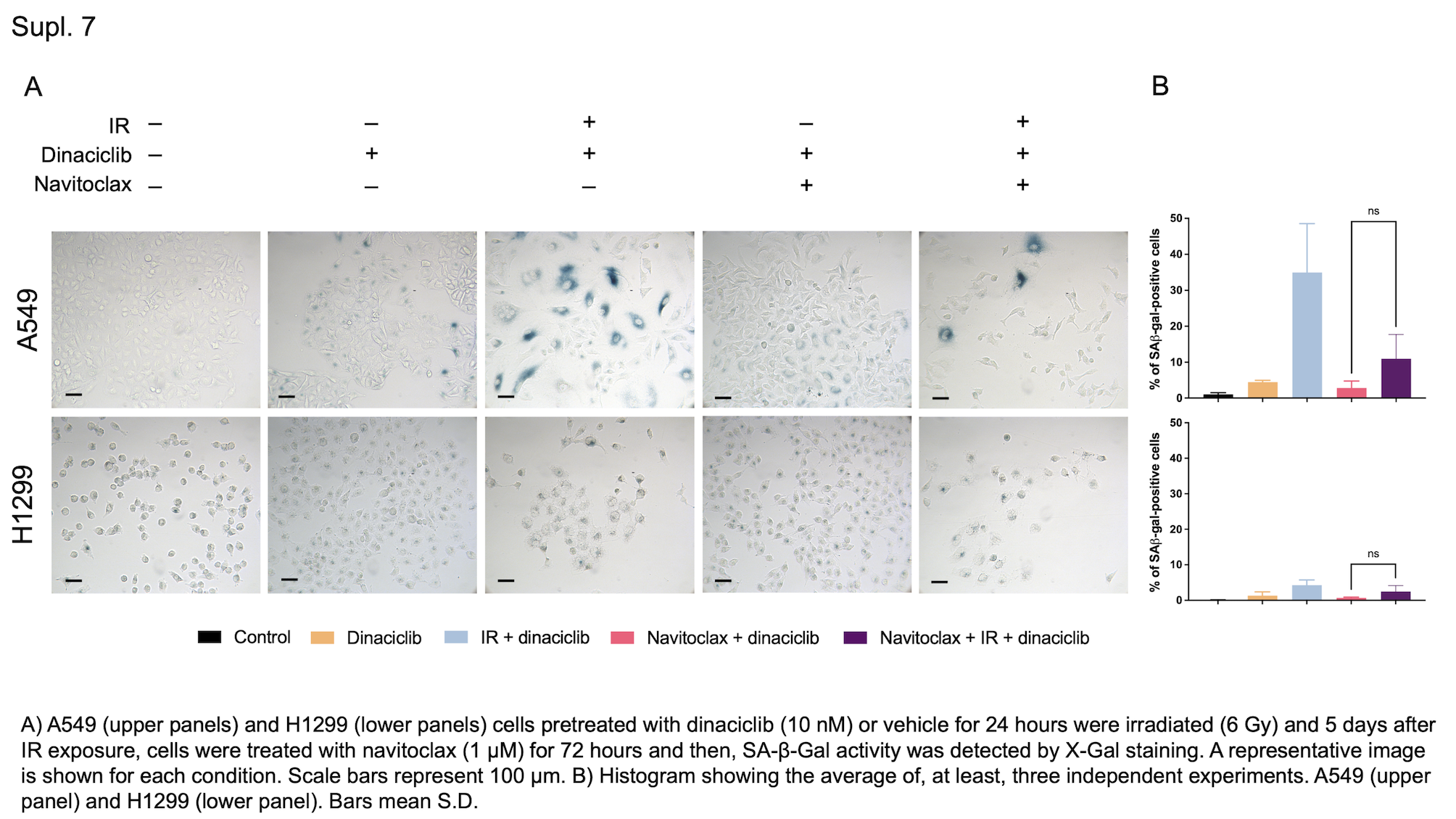

Supplement: Supplementary file 7 — Fig. S7. SA‐β‐Gal activity detection in the presence/absence of navitoclax treatment. [file MOL2-19-1265-s011.tiff]

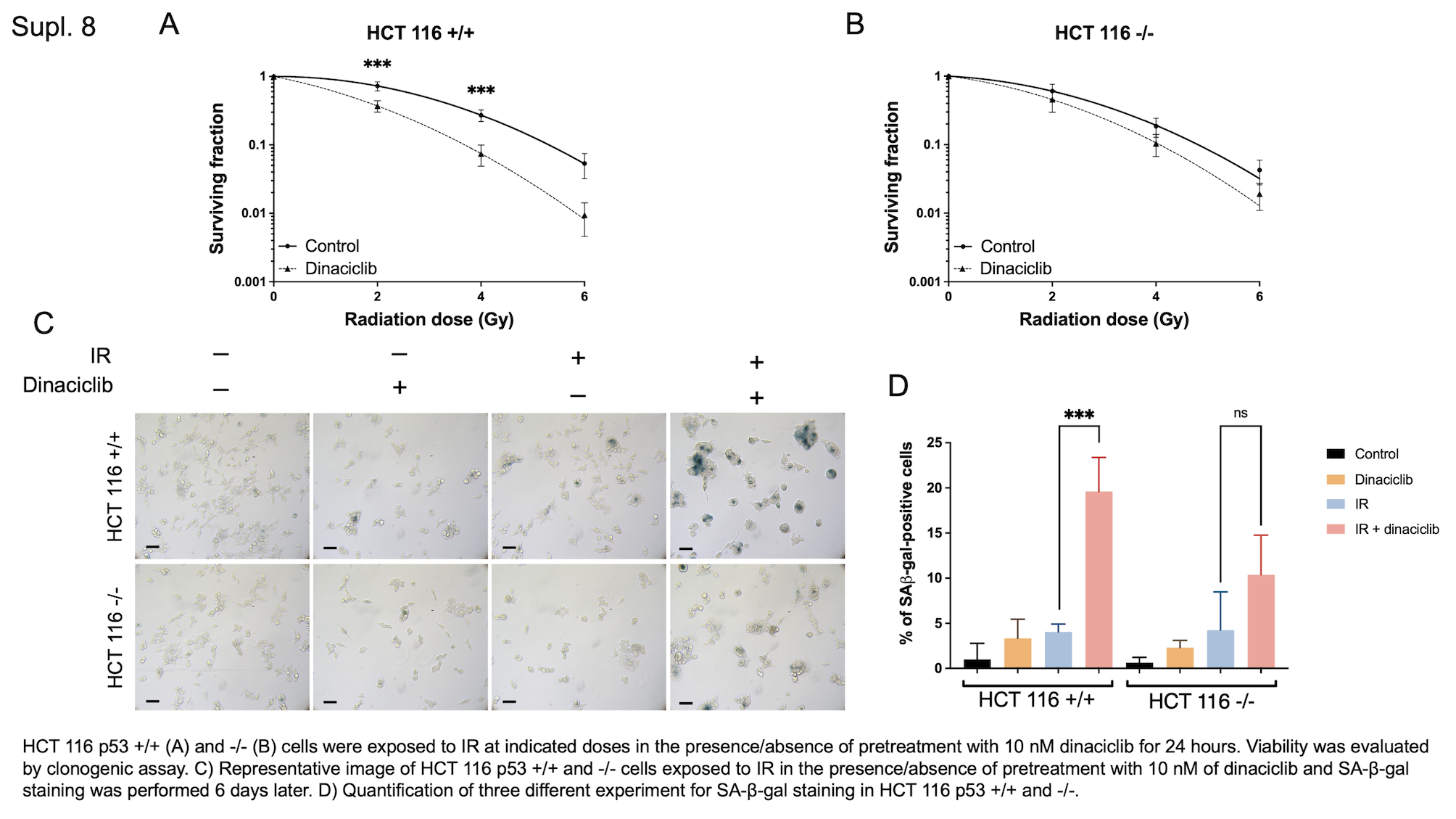

Supplement: Supplementary file 8 — Fig. S8. Study of dinaciclib effects in the isogenic c model of HCT 116 (HCT 116 p53 +/+ and −/). [file MOL2-19-1265-s013.tiff]

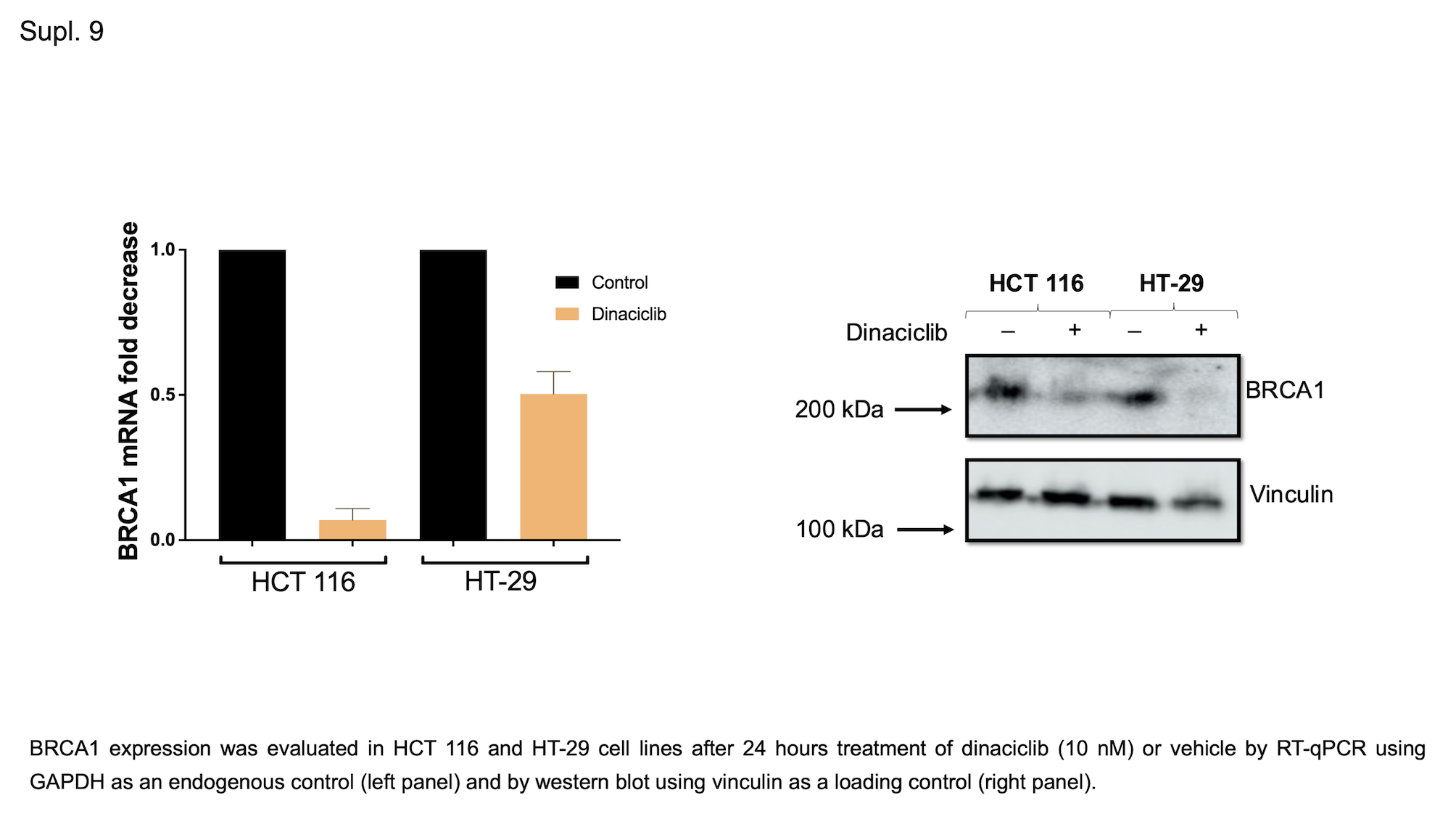

Supplement: Supplementary file 9 — Fig. S9. Effect of dinaciclib onto BRCA1 expression in HCT 116 and HT‐29 cell lines. [file MOL2-19-1265-s016.tiff]

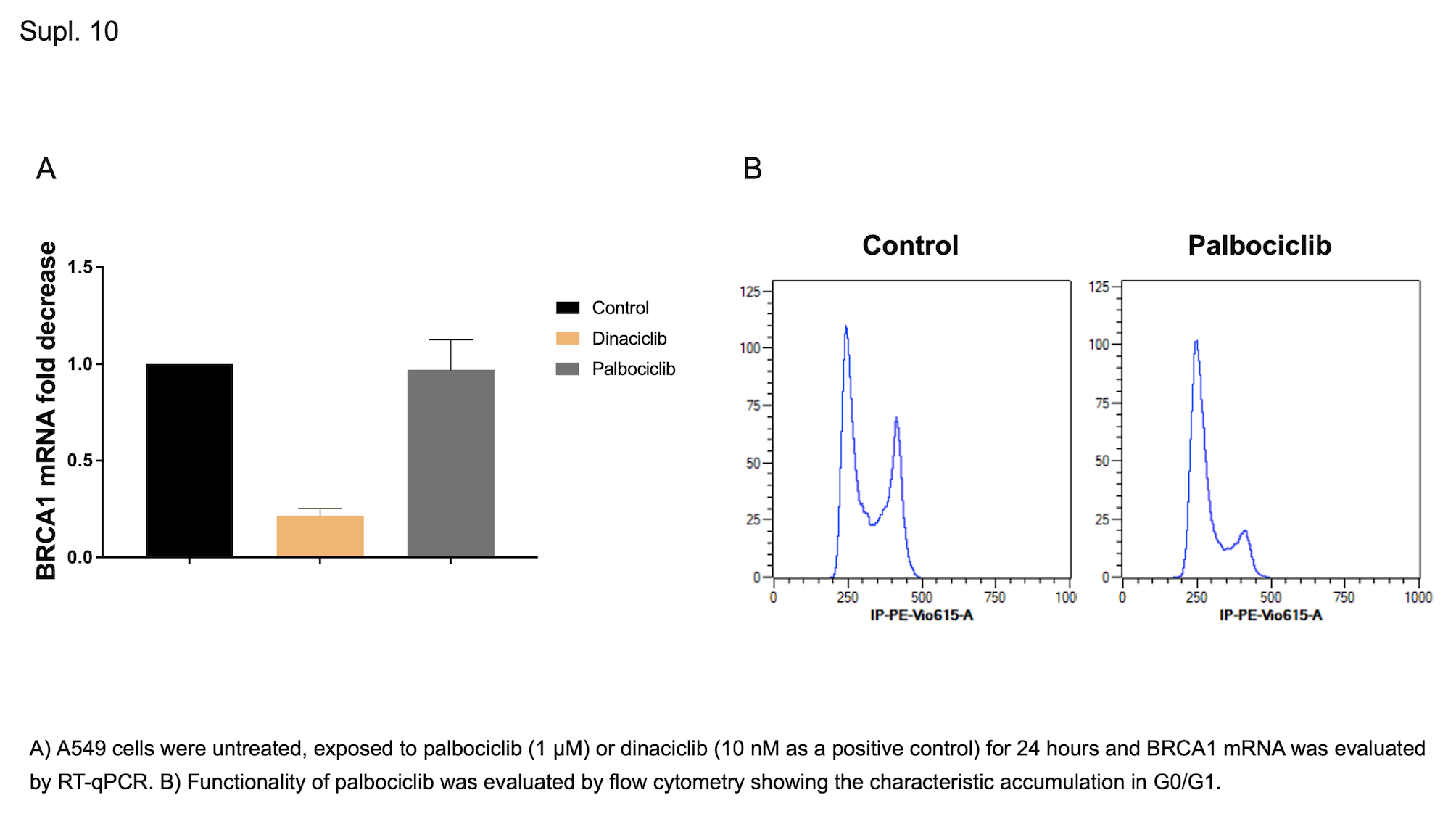

Supplement: Supplementary file 10 — Fig. S10. Effect of palbociclib onto BRCA1 expression in A549 cell line. [file MOL2-19-1265-s007.tiff]

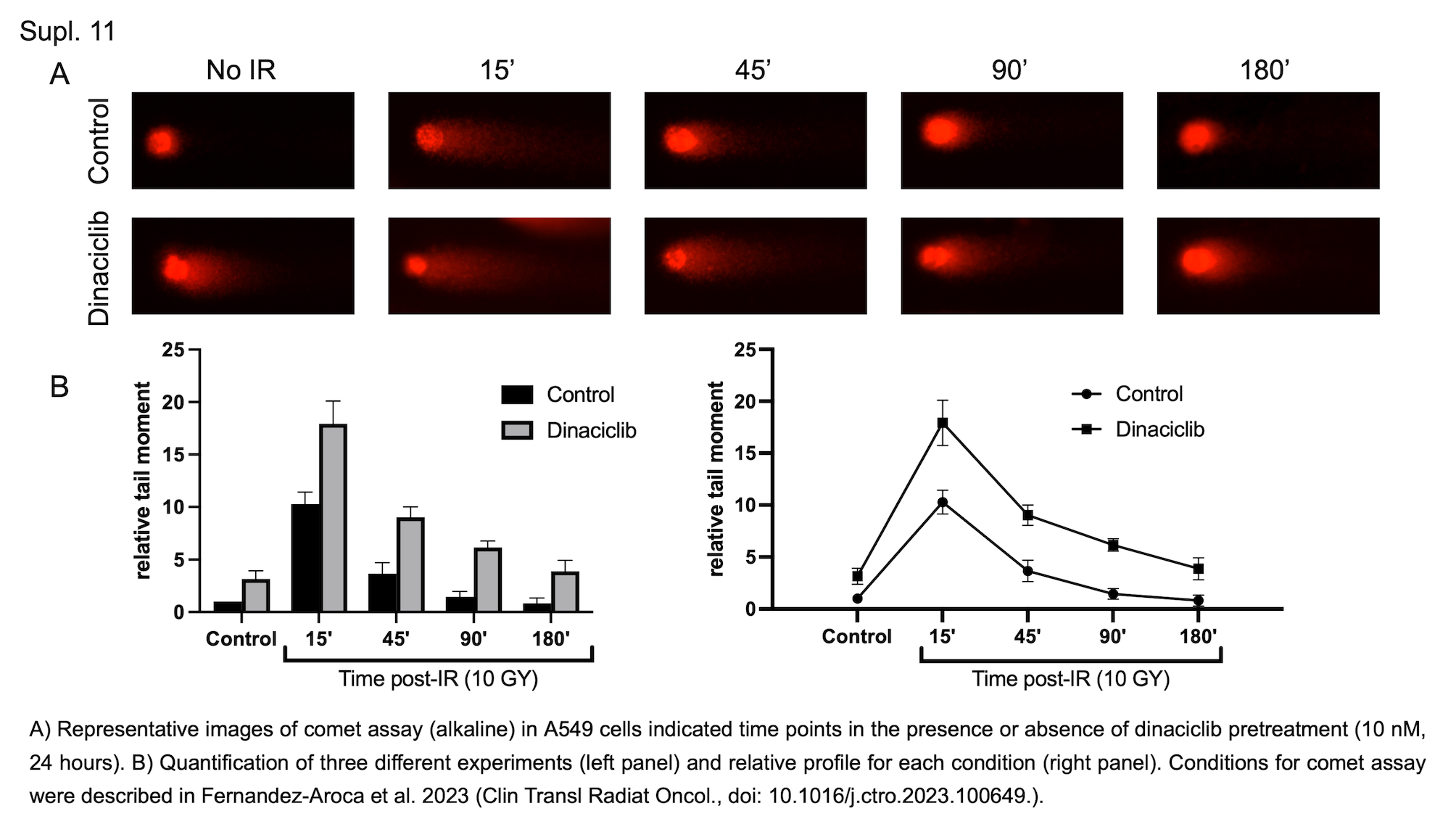

Supplement: Supplementary file 11 — Fig. S11. Comet assay in A549 cells with or without dinaciclib pretreatment. [file MOL2-19-1265-s010.tiff]

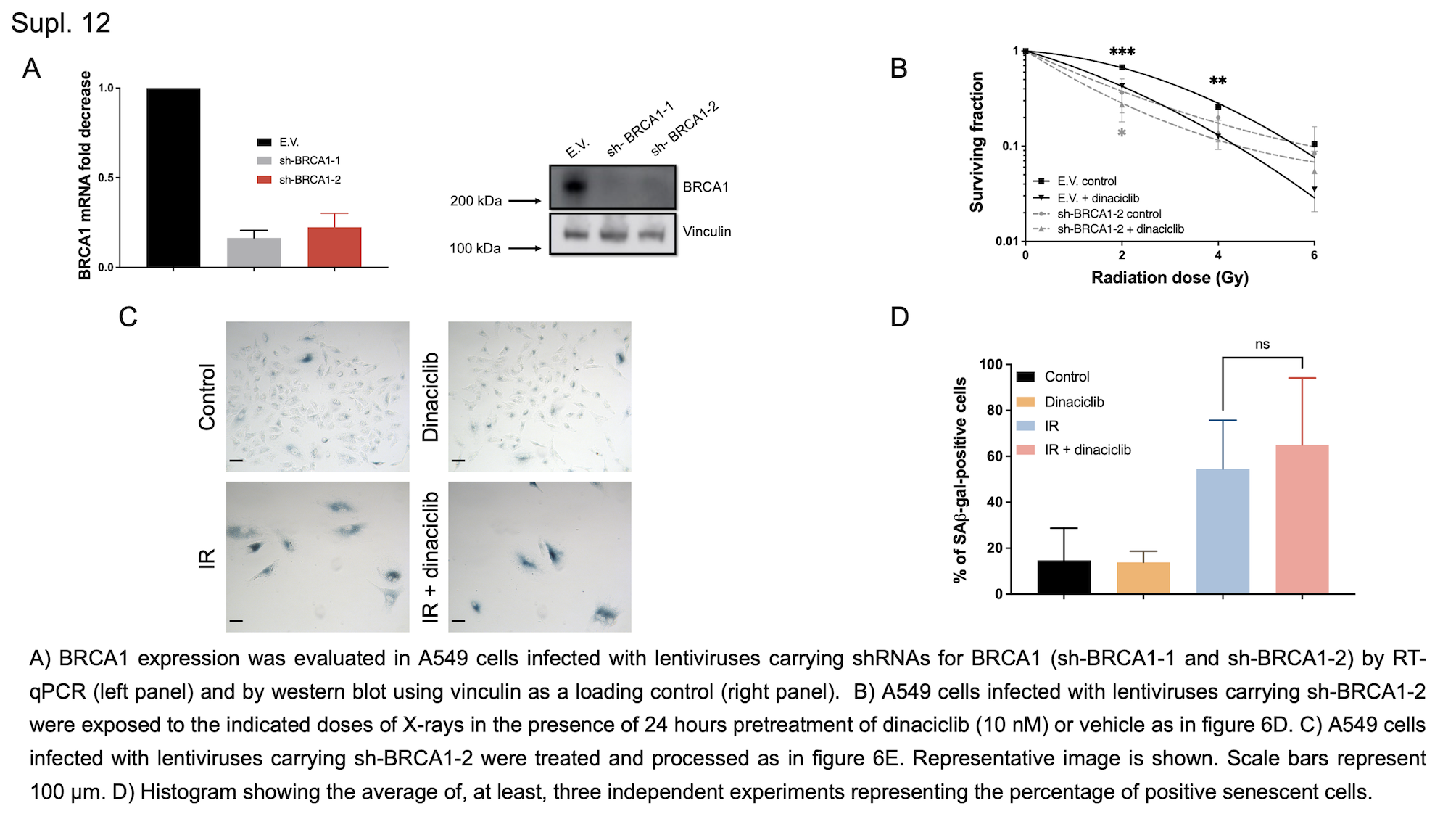

Supplement: Supplementary file 12 — Fig. S12. Effect of BRCA1 interference (sh‐BRCA1‐2) on dinaciclib‐associated radiosensitivity in the A549 cell line. [file MOL2-19-1265-s015.tiff]

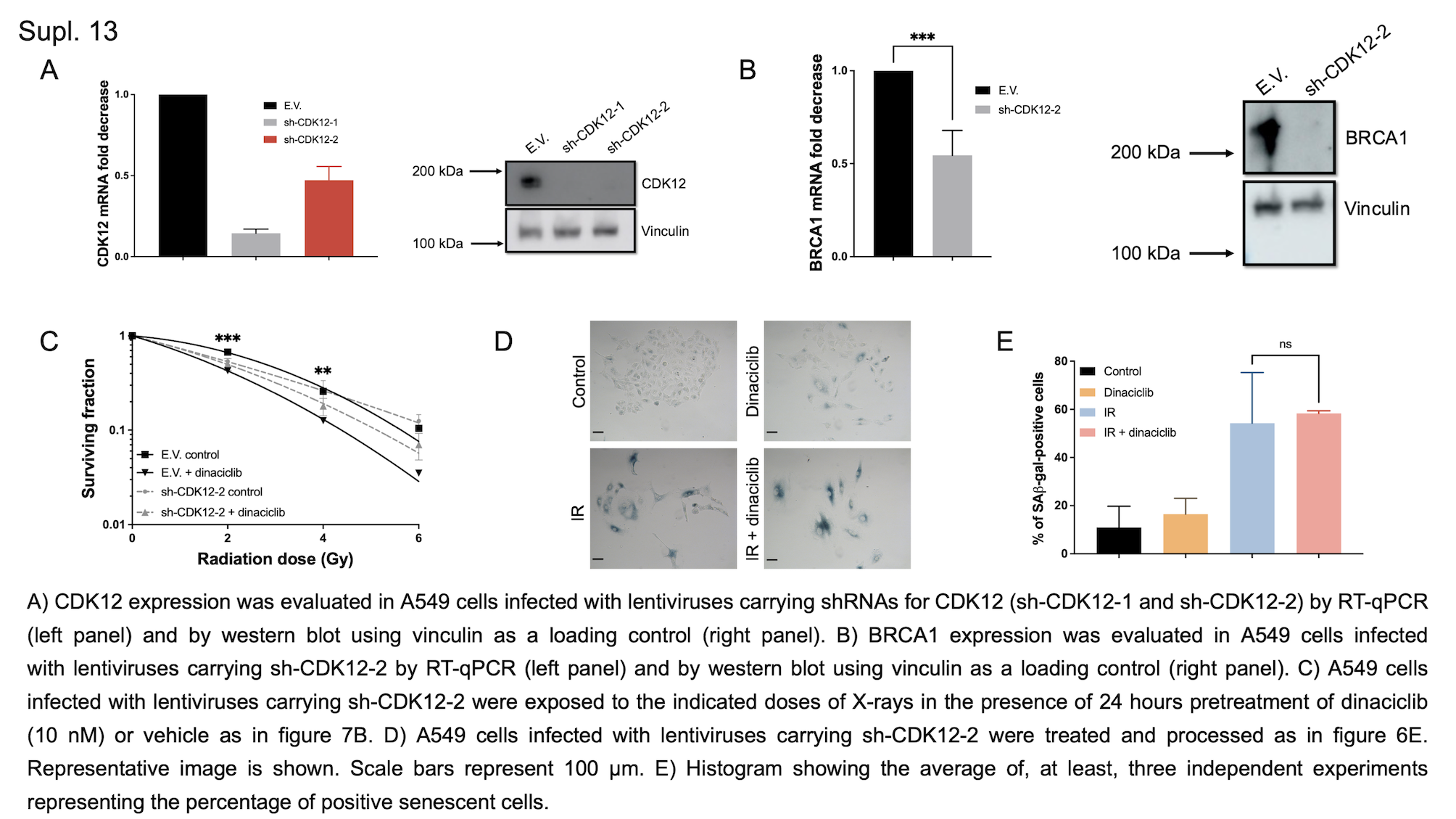

Supplement: Supplementary file 13 — Fig. S13. Effect of CDK12 interference (sh‐CDK12‐2) on dinaciclib‐associated radiosensitivity in the A549 cell line. [file MOL2-19-1265-s017.tiff]

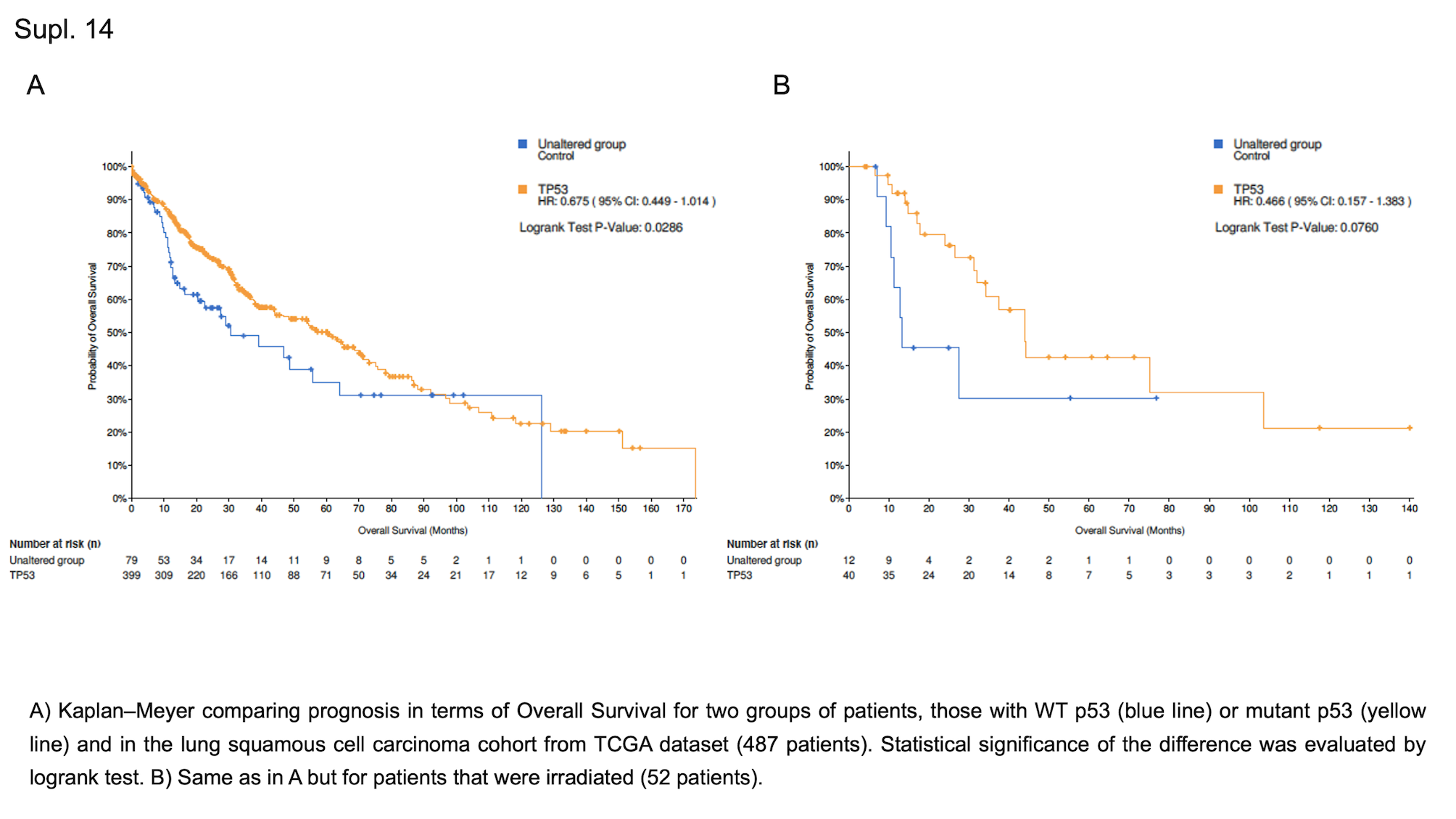

Supplement: Supplementary file 14 — Fig. S14. In silico study of TP53 mutation in the overall survival of the lung squamous cell carcinoma cohort from TCGA. [file MOL2-19-1265-s004.tiff]

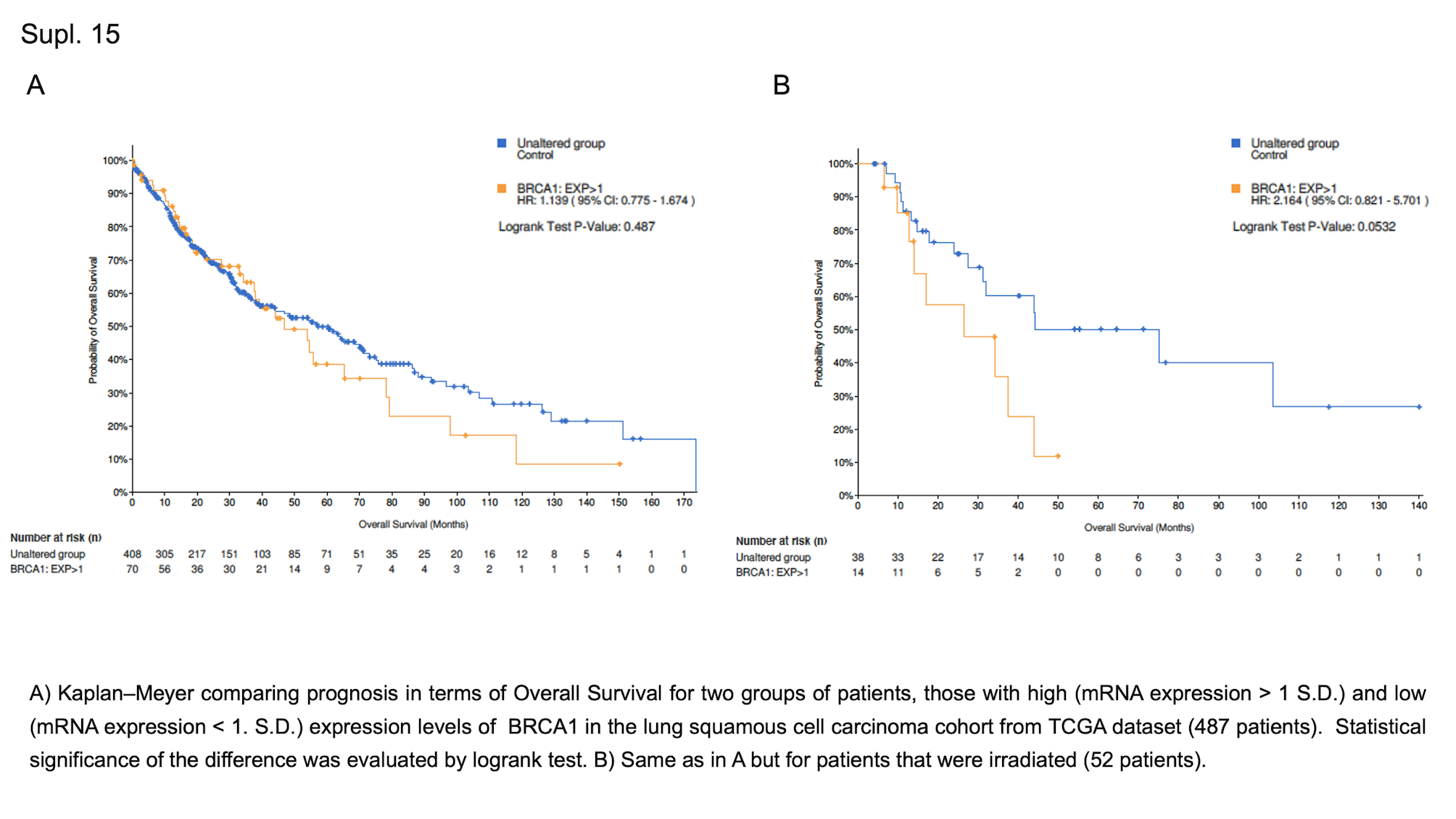

Supplement: Supplementary file 15 — Fig. S15. In silico study of BRCA1 expression (mRNA) in the overall survival of the lung squamous cell carcinoma cohort from TCGA. [file MOL2-19-1265-s003.tiff]
