# Supplementary material for: The CDK12–BRCA1 signaling axis mediates dinaciclib‐associated radiosensitivity through p53‐mediated cellular senescence
Source: Mol Oncol. 2024 Dec 3;19(4):1265–80. doi: 10.1002/1878-0261.13773 (PMC11977655; doi:10.1002/1878-0261.13773)
Supplement: Supplementary file 16 — Table S1. List of antibodies used. [file MOL2-19-1265-s012.docx]

**Supplementary table 1.** **List of antibodies used**

| **Use** | **Antibody** | **Dilution** | **Manufacturer** | **Reference** |
| --- | --- | --- | --- | --- |
| Immunocytochemistry | p-H2AX | 1:500 | Cell Signaling Technology | 9718 |
| Immunocytochemistry | Anti-rabbit Alexa Fluor 488 | 1:2000 | Invitrogen | A-11008 |
| Western Blot | P53 | 1:500 | Santa Cruz Biotechnology | sc-47698 |
| Western Blot | P21 Waf1/Cip1 (12D1) | 1:1000 | Cell Signaling Technology | 2947 |
| Western Blot | GAPDH (14C10) | 1:1000 | Cell Signaling Technology | 2118 |
| Western Blot | BRCA1 | 1:1000 | Cell Signaling Technology | 9010 |
| Western Blot | KAP1 | 1:1000 | Bethyl Laboratories | A300-274A-T |
| Western Blot | p-KAP1 | 1:1000 | Bethyl Laboratories | A700-013-T |
| Western Blot | CDK12 | 1:1000 | Cell Signaling Technology | 11973 |
| Western Blot | Tubulin | 1:2000 | Santa Cruz Biotechnology | sc-32293 |
| Western Blot | Vinculin | 1:3000 | Sigma-Aldrich | V9264 |
| Western Blot | Anti-mouse-HRP | 1:2000 | Cell Signaling Technology | 7076 |
| Western Blot | Anti-rabbit-HRP | 1:2000 | Cell Signaling Technology | 7074 |
