# Supplementary material for: The CDK12–BRCA1 signaling axis mediates dinaciclib‐associated radiosensitivity through p53‐mediated cellular senescence
Source: Mol Oncol. 2024 Dec 3;19(4):1265–80. doi: 10.1002/1878-0261.13773 (PMC11977655; doi:10.1002/1878-0261.13773)
Supplement: Supplementary file 17 — Table S2. List of primers used. [file MOL2-19-1265-s001.docx]

**Supplementary table 2 List of primers used**

| **Use** | **Target** | **Forward** | **Reverse** |
| --- | --- | --- | --- |
| RT-qPCR | *GAPDH* | TCGTGGAAGGACTCATGACCA | CAGTCTTCTGGGTGGCAGTGA |
| RT-qPCR | *IL-1β* | TGCACGTCCGGGACTCACA | CATGGAGAACACCACTTGTTGCTCC |
| RT-qPCR | *IL-6* | GATGAGTACAAAAGTCCTGATCC | CTGCAGCCACTGGTTCTGT |
| RT-qPCR | *IL-8* | AGACAGCAGAGCACACAAGC | ATGGTTCCTTCCGGTGGT |
| RT-qPCR | *BRCA1* | TAGGGCTGGAAGCACAGAGT | AATTTCCTCCCCAATGTTCC |
| RT-qPCR | *CDK12* | TGAAAACCCAAGAGCCAGCA | GTGGAAGAATGTGAGGAGGACAT |
| RT-qPCR | *TP53* | TCCTGCCATTTTGGGTTT | GCAGGCCAACTTGTTCAGTG |
